# Transition-metal-mediated reduction and reversible double-cyclization of cyanuric triazide to an asymmetric bitetrazolate involving cleavage of the six-membered aromatic ring†

**DOI:** 10.1039/d0sc04949b

**Published:** 2020-12-08

**Authors:** Shivaiah Vaddypally, Vitaly G. Kiselev, Alex N. Byrne, C. Franklin Goldsmith, Michael J. Zdilla

**Affiliations:** Temple University 1901 N. 13^th^ St. Philadelphia PA 19122 USA mzdilla@temple.edu; School of Engineering, Brown University 184 Hope St. Providence RI 02912 USA franklin_goldsmith@brown.edu; Novosibirsk State University 1 Pirogova Str. 630090 Novosibirsk Russia; Institute of Chemical Kinetics and Combustion SB RAS 3 Institutskaya Str. 630090 Novosibirsk Russia; Semenov Federal Research Center for Chemical Physics RAS 4 Kosygina Str. 119991 Moscow Russia

## Abstract

Cyanuric triazide reacts with several transition metal precursors, extruding one equivalent of N_2_ and reducing the putative diazidotriazeneylnitrene species by two electrons, which rearranges to *N*-(1′*H*-[1,5′-bitetrazol]-5-yl)methanediiminate (biTzI^2−^) dianionic ligand, which ligates the metal and dimerizes, and is isolated from pyridine as [M(biTzI)]_2_Py_6_ (M = Mn, Fe, Zn, Cu, Ni). Reagent scope, product analysis, and quantum chemical calculations were combined to elucidate the mechanism of formation as a two-electron reduction preceding ligand rearrangement.

## Abstract 2

Cyanuric triazide reacts with transition metal precursors, extruding N_2_ and reducing the ligand by two electrons, which breaks an aromatic ring and rearranges to a bitetrazolylmethanediiminate (biTzI^2−^) ligand, forming two new aromatic rings.

## Graphical abstract

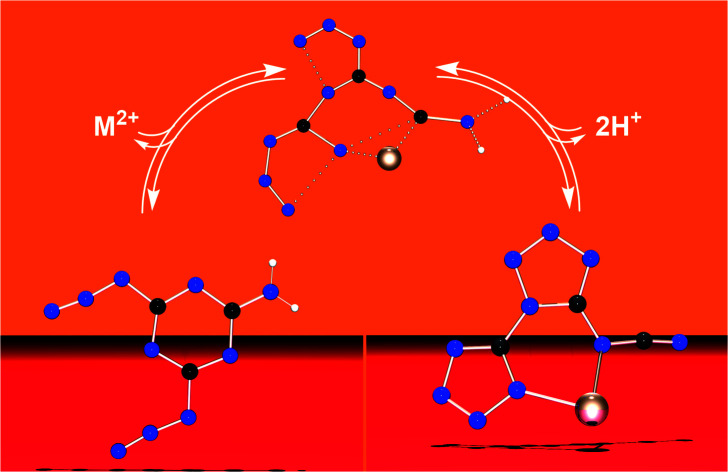

## Introduction

Novel cycloaddition reactions are of fundamental interest to synthetic chemistry, as (hetero)cyclic compounds play important roles in tuning stability,^1–12^ electronic properties,^13–15^ and biological activity of molecules.^16–20^ Access to specific or novel approaches for cyclization are of fundamental interest for enhancing the synthetic chemist's toolbox. Nitrogen-rich heterocycles are of fundamental interest in synthetic chemistry as biological mimics and pharmaceuticals,^13,16–20^ and as energetic materials such as explosives and propellants.^21–26^ In this latter category, heterocyclization reactions have been crucial to the realization of structures with improved kinetic stability and physical properties.^1–12,21–26^

Among the most important cycloadditions is the so-called Huisgen [2 + 3] cycloaddition, which results in the joining of reactive and (frequently) impact sensitive azides with reactive alkynes into kinetically stable five-membered 1,2,3-triazole rings.^27–29^ Azole based systems are of interest as novel high explosives due to the remarkable stability of the five-membered heteroaromatic ring, which makes this class of molecules generally more insensitive than energetic materials with comparable nitrogen content.^21,30,31^ The original Huisgen cycloaddition is generally agreed to be a concerted [3 + 2] cycloaddition that gives two possible stereochemical outcomes. The copper-catalyzed variant, discovered approximately at the same time by Meldal^32^ and Sharpless,^33^ operates by a different mechanism (nicknamed the “click” reaction due to its success in selective biorthogonal chemistry), yielding exclusively the 1,4-substituted product. Azides may also be cyclized with nitriles to give the four-nitrogen tetrazole aromatic ring.^23^

Other cycloadditions of azide reactions are of interest as well. Our group^34,35^ and others^36–41^ have explored the cycloaddition of two azide units, coupled to metal-mediated extrusion of N_2_, to give five-membered tetrazene metallacycles. In these complexes, a redox-non-innocent four-nitrogen chain of catenated nitrogen ligands acts as a bidentate ligand.^34,35,39–41^ A highly relevant cycloaddition reaction of azide occurs on *ortho*-azidoazines, *viz.*, nitrogen-substituted benzene rings with an azide group ortho to a ring nitrogen. This species can undergo a reversible isomerization to a indole-like fused bicycle containing a tetrazole ring fused to the six-membered azine.^3,7,42–46^ Oligomerized small molecules with high-nitrogen–carbon ratio are of interest in astrochemistry, where small molecules such as nitrogen and HCN in deep space may be activated by high energy radiation and aggregate into complex organic molecules.^47^ Here we report a related double cycloaddition rearrangement reaction of cyanuric triazide (2,4,6-triazido-1,3,5-triazine, 1) following reduction of the substrate to release N_2_, concomitant with the cleavage of the 6-membered ring to give *N*-([1,5′-bitetrazol]-5-yl)methanediiminate ligand in a dimeric metal complex. This asymmetrically linked bitetrazole nucleus is uncommon, limited to a few reports with structurally characterized examples: one with amino and nitramido side chains,^48^ and one on metal complex chemistry of the parent bitetrazole.^49^ The variant reported in this paper contains a pendant carbodiimide group bridging two metal centers. Upon demetallation, this ligand undergoes a similar reverse reaction to regenerate the azidotriazine ring.

## Results and discussion

**Scheme 1:**
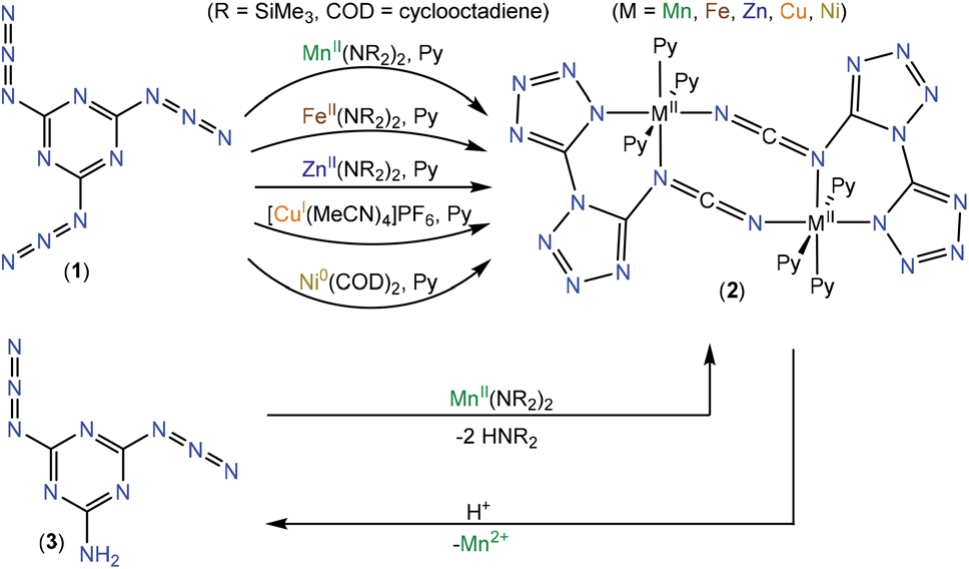
Metal mediated conversions between azidotriazenes and metal–biTzI complexes.

**Fig. 1:**
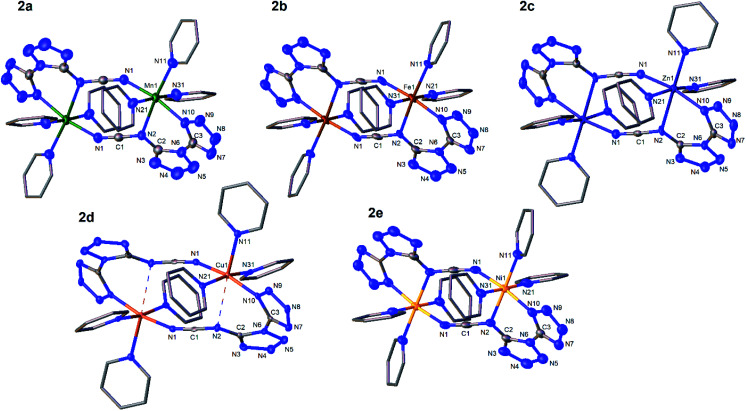
Thermal ellipsoid plots of [M^II^(biTzI)Py_3_]_2_ (2). M = Mn (2a), M = Fe (2b), M = Zn (2c), M = Cu (2d), M = Ni (2e). Ellipsoids set at 50% probability. Pyridine carbon atoms are shown in stick mode, and hydrogen atoms and lattice solvents omitted for clarity.

**Scheme 2:**
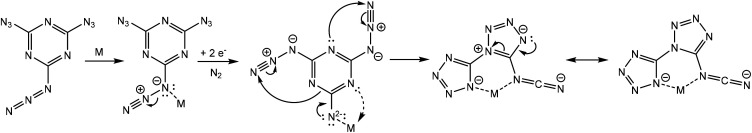
Proposed mechanism of metal mediated reaction/rearrangement of 1 into biTzI ligand.

We intended to extrapolate our work on the formation of metallotetrazene species^34–41^ from addition of azides at manganese centers to cyanuric triazide^50^ by reaction with Mn(NR_2_)_2_ ^51^) (R = SiMe_3_). However, this experiment resulted in an entirely different type of chemical reaction. Single-crystal X-ray diffraction analysis of the crystalized product of this reaction revealed the structure to be that of a dimeric metal complex consisting of two dibasic *N*-([1,5′-bitetrazol]-5-yl)methanediiminate ligands (biTzI), two Mn^II^ ions, and capping pyridine ligands. The resulting product, [Mn(biTzI)Py_3_]_2_ (2a) is shown in Scheme 1 and Fig. 1. The drastic ligand rearrangement resulting in the loss of the six-membered aromatic ring and the appearance of two new five-membered aromatic heterocycles was unexpected, but may be rationalized by a ligand reduction to extrude N_2_, accompanied by a rearrangement of the resulting imido species involving N–C cleavage, and closure of the azides with *ortho* azine nitrogen atoms (Scheme 2). This isomerization of a sensitive cyanuric polyazide into more stable tetrazole ring systems represents a novel transformation into a new type of energetic moiety: one involving the cleavage of a six member aromatic ring, which is an exceedingly rare phenomenon usually discussed in the context of the 2-aminophenol dioxygenase enzyme^52^ and model chemistry thereof.^53,54^

However, charge counting considerations lead to a formal oxidation state of 2+ on the manganese ions, suggesting that the metal ion has not been oxidized. We initially hypothesized that additional equivalents of Mn^II^ act as a sacrificial reductant to form Mn^III^ or Mn^IV^, and that the resulting reduced ligand framework then assembles with additional equivalents of Mn^II^ to generate 2. However, we were unable to identify any other byproducts of the reaction containing oxidized manganese. As a test of the hypothesis that the metal serves two roles (reductant and divalent metal source) we performed the reaction with a more easily oxidized metal ion, Fe^II^, using the analogous Fe(NR_2_)_2_ ^51^ reagent, which is highly reducing and favors formation of Fe^III^. However, the isolated product of this reaction was the analogous ferrous complex [Fe(biTzI)Py_3_]_2_ (2b) also containing formal Fe^II^ ions (Scheme 1, Fig. 1). Again, we were unable to isolate any oxidized iron species from this reaction.

Challenged by the question of the origin of the reducing equivalents, we considered next the synthesis of an analog of 2 using a strictly redox innocent metal, Zn^2+^, an approach that should entirely preclude metal-based redox reactions. To our surprise, the reaction of Zn(NR_2_)_2_ with cyanuric triazide resulted in the isolation of the analogous zinc complex, [Zn(biTzI)Py_3_]_2_ (2c) in good yield, and which comprised the same two-electron reduced biTzI ligand complexed to Zn^II^ (Scheme 2). In addition to crystallographic characterization, the diamagnetic 2c could be characterized by ^13^C NMR (other compounds experience paramagnetic broadening of resonances and are thus invisible). The NMR spectrum shows three large signals corresponding to pyridine ligands, and three small signals corresponding to quaterinary carbons, with the carbodiimide signal shifted significantly upfield to 119 ppm due to its location in two shielding cones of the C=N double bonds (Fig. S4†).

Inspired by the successful synthesis of an analog of 2 with a redox-innocent metal, we considered the possibility that the ligand reduction is not a prerequisite for ligand rearrangement, and whether instead a pathway involving a rearrangement of the ligand prior to reduction (for example, by adventitious oxidation of solvent) could result in product formation. To address this question, we performed quantum chemical calculations using modern, reliable DLPNO-CCSD(T)^55,56^ and CCSD(T)-F12 methodologies^57,58^ along with the polarized continuum model (PCM)^59^ to account for the solvent. We considered several possibilities for the rearrangement chemistry: (1) the extrusion of N_2_ to generate either singlet or triplet neutral nitrene, followed by a ligand rearrangement. (2) one-electron reduction with the extrusion of N_2_ to generate an anionic nitrenyl radical, or (3) a two-electron reduction to extrude N_2_ and generate a metal–ligated imido species.

The mechanism of (1) is disfavored based on theoretical considerations, as the activation barrier for two types of cyclization rearrangements is high: *viz.*, the formation of the double-cyclized product (without a cleavage of the six-membered ring) has an effective activation barrier around 31 kcal mol^−1^ (this corresponds to a reaction time *t*_rxn_ ∼30 years). Moreover, the activation barrier of the thermal extrusion of N_2_ yielding a nitrene intermediate, is predicted to be 38 kcal mol^−1^, and the subsequent reaction cascade did not favor the formation of the biTzI ligand system or a similar species (see Fig. S1†).

The one-electron reduction of 1 renders the N_2_ elimination yielding a nitrenyl radical anion to be an extremely fast process (in agreement with the literature data for similar species,^60^ the activation barrier is close to zero, Fig. S2†). The latter compound has a lower-barrier entrance channel to the rearrangement chemistry of only 16 kcal mol^−1^, which could cascade in several steps to the biTzI˙^−^ anion radical, whose formation is exothermic by 9 kcal mol^−1^ (Fig. S2†). However, this is not the most stable rearrangement product in the cascade; a bis(tetrazolo)[1,5-*a*:5′,1′-*d*][1,3,5]triazinyliminyl radical anion is *ca.* 5 kcal mol^−1^ more favorable thermodynamically than the latter species (Fig. S2†).

**Fig. 2:**
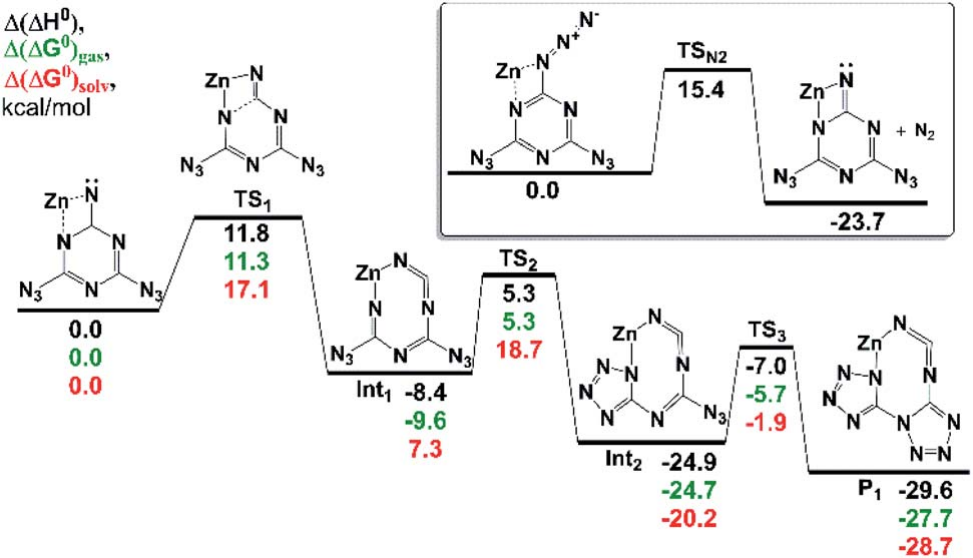
The stationary points on the PES corresponding to the rearrangements of Zn^2+^ complex with a dianion intermediate. Inset: the N_2_ elimination from the initial complex of Zn and 1. All values are calculated at the DLPNO-CCSD(T)/aVQZ//M06-2X/6-311++G(2df,p) level of theory and are given in kcal mol^−1^. The PCM free energies of solvation are calculated at the same DFT level of theory using tetrahydrofuran as a solvent.

The most favorable pathway of 1 transformation involves a two-electron reduction preceding a molecular nitrogen elimination followed by a rearrangement of the nitrene intermediate. The examination of an N_2_-eliminated dianionic imide coordinated to a model Zn^2+^ center predicted a favorable rearrangement to the expected [biTzI]^2−^ ligand product in a sequence of three irreversible reactions (Fig. 2). The last rate-limiting step occurring *via* TS_3_ has an activation barrier ∼18 kcal mol^−1^, which is affordable at room temperature (the reaction time of a few seconds). The lowest-energy product predicted in the cascade, is exothermic by 29.6 kcal mol^−1^ (Fig. 2), and represents an essentially identical mechanism to that proposed in Scheme 2, proceeding in three low-barrier steps.

As a further test of the proposed mechanism where reduction precedes a ligand rearrangement, we synthesized 2a using the already-reduced variant of 1: *viz.*, 2-amino-4,6-diazido-1,3,5-triazine (3).^61^ This precursor was combined with Mn(NR_2_)_2_ in pyridine to determine whether simple deprotonation of already-reduced ligand 3 by protolysis with 1 would result in the proposed ligand arrangement. Indeed, this reaction resulted in the isolation of 2a, which was confirmed by single-crystal X-ray crystallographic analysis. To our surprise, the attempt to demetallate 1a by acid hydrolysis resulted not in the isolation of (biTzI)H_2_, but in a reverse rearrangement of the hydrolyzed ligand back to 3 (Scheme 1) which was isolated and identified by single-crystal XRD (Fig. S16†). This result suggests that the six-membered-ring aryl bond cleavage and rearrangement to the bitetrazole-based [biTzI] ligand is favored in basic media, but is reversible and may be converted back by cleavage of the two tetrazole rings and reformation of the six-membered azine ring in acidic media (Scheme 1).

Given the predicted favorableness of the ligand reduction preceding rearrangement, we considered the hypothesis that trimethylsilylamido (NR_2_^−^) ligands of the precursor M(NR_2_)_2_ could act as the reductant, undergoing a reductive elimination and forming tetrakis(trimethylsilylamido)hydrazine (R_2_N–NR_2_) as a byproduct. The latter compound is isolable, stable, and crystalline,^62–64^ and its formation is plausible due to the known redox relationships between amines/amides and hydrazine.^65^ R_2_N–NR_2_ has a reported ^1^H NMR upfield chemical shift of 0.2 ppm in benzene-d_6_ solvent, distinct from the chemical shifts of hexamethyldisiloxane (0.12)^66^ and hexamethyldisilazane (0.10).^67^ The analysis of the crude reaction mixture of the synthesis of 2c reveals the formation of R_2_N–NR_2_, (Fig. S3†) and provides an explanation for the observed redox processes, and implies the following reaction stoichiometry:1
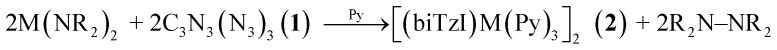
With support for a mechanism of reduction of 1 preceding ligand rearrangement, we sought to answer whether this reaction could be achieved using one- or two-electron metal-based redox chemistry instead of the above described ligand-based redox chemistry. As compounds 2a–2c suggest this ligand promotes stabilization of the 2+ oxidation state for mid-to-late transition metals, we selected reducing metal precursors at lower, more reducing oxidation states with redox innocent counterions: namely, copper(i) hexafluorophosphate tetraacetonitrile affords the opportunity to test a one-electron metal-based reductant for the formation of 2. Reaction of this compound with 1 affords [Cu(biTzI)Py_3_]_2_ (2d), analogous to 2a–c, except for a significant distortion of the octahedral ligand field, with a more distal axial pyridyl-*N*-Cu distance of ∼2.28 Å (*vs.* ∼2.07 Å for the equatorial pyridyl-*N*-Cu distances) and a very distant imino-*N*-Cu axial contact distance of 2.75 Å (*vs.* 1.97 Å for the equatorial imino-*N*-Cu bond). This deviation from the octahedral lilgand field is consistent with the expected Jahn–Teller distortion for the d^9^ electron configuration of Cu^II^, and the preference of Cu^II^ for lower coordination numbers (see Table S1†). The generation of the Cu^II^ oxidation state suggests a one electron oxidation of the metal center, though the ligand has still reduced by two electrons to generate [biTzI]^2−^, suggesting the involvement of a two additional Cu^I^ equivalents that act as additional reductant, and which would be removed as a byproduct hexafluorophosphate salt:2
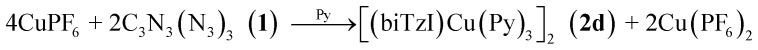


Finally, in order to test for the generation of a variant of 2 with a metal-based two-electron reductant, we tested the reaction of the strong reducing agent bis(cyclooctadiene)nickel(0) (COD_2_Ni^0^) with 1. The expected product [Ni(bTzNCN)Py_3_]_2_ (2e) is isolated in good yield according to eqn (3):3
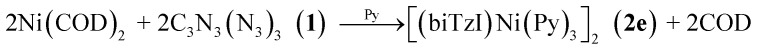


The scope of synthetic chemistry including the results from quantum chemical calculations indicate that the reduction of 1 is favorable if coupled to the described cycloaddition rearrangement, and can be mediated by metal based reduction (both one- and two-electron reductants) as well as by ligand-based reduction mediated at redox innocent transition metals.

## Experimental and computational details

### Precautions

The low-carbon azide and tetrazole-based molecules presented in this report are explosive materials. They should be stored in solution or isolated only in quantities of a few milligrams. Shock, spark, heat from exposure to air can result in explosion. All syntheses were performed in microscale for maximum safety. Proper precautions should be taken, including use of standard laboratory PPE plus explosion proof mask, shield, Kevlar gloves, use of plastic spatulae, and electrical grounding while handling these materials. While we never experienced any accidental initiation of compounds 2a–2e resulting from physical stimuli (*e.g.*, spatula scraping), appropriate caution is recommended. Detonation was, however, observed in these materials when irradiated by a pulsed laser.

#### General

All manipulations were performed under a rigorous dry, anaerobic atmosphere of nitrogen gas using standard glove box and Schlenk line techniques. Acetonitrile and pentane were purified using an Innovative Technology, Inc. Pure Solv.™ system. Tetrahydrofuran and pyridine were distilled from sodium benzophenone ketyl under a nitrogen atmosphere. Ni(COD)_2_, Zn(NR_2_)_2_ (R = SiMe_3_), tetrakis(acetonitrile)copper(i) hexafluorophosphate and ^*t*^BuNH_2_, were purchased from commercial vendors (Aldrich, Strem). Reagents were used without further purification. Anhydrous solvents were used throughout all experiments. FT-IR spectra were recorded in the range of 400–4000 cm^−1^ on a Nicolet iS5 spectrometer with a Nicolet iD5 ATR attachment. UV-visible spectra were recorded on a Shimadzu UV-1800 UV spectrophotometer in the range of 200–900 nm. Single crystal X-ray diffraction were performed on a Bruker KAPPA APEX II DUO diffractometer. Single crystals were mounted on a MiTeGen loop using Apiezon-N grease. NMR spectra were obtained on a Bruker AV-III 500 MHz NMR spectrometer. CHN combustion analyses were attempted on these compounds, but reproducible results were not obtained (even in the same sample preparation), suggesting incomplete combustion. We find this a common problem with metal-containing energetic materials, likely due to incomplete combustion during explosion and formation of metal nitrides. As such, quantitative purity assessment is not available, though these procedures produce high-quality crystals that index to the same unit cell reproducibly.

### Synthesis

Mn(NR_2_)_2_(THF)_2_,^51^ Fe(NR_2_)_2_(THF)_2_,^51^ (R = SiMe_3_), cyanuric triazide (1),^50^ were prepared according to literature protocol.

#### 2-Amino-4,6-diazido-1,3,5 triazine (3)

3 was prepared using a literature protocol^61^ and identified using X-ray crystallography and ^1^H NMR spectroscopy. Unit cell: triclinic, *a* = 8.018(4) Å *b* = 4.669(3) Å *c* = 18.877(10) Å, *α* = 72.463(4)°, *β* = 101.238(10)°, *γ* = 71.422(3)° *V* = 693.1(7) Å^3^. ^1^H NMR [ppm] (500 MHz, C_6_D_6_): *δ*(ppm): 8.00 (NH_2_).

#### [(biTzI)Mn(Py)_3_]_2_ (2a)

##### Method A

0.10 g (0.15 mmol) of Mn(NR)_2_(THF)_2_ is dissolved in tetrahydrofuran followed by addition of 0.065 g (0.32 mmol) of cyanuric triazide (1). After dissolving these two reactants 200 μL of tBuNH_2_ was added, which results in a color change to red. This reaction mixture is stirred for one hour and the solvent is removed *in vacuo*. The dried product is washed with pentane and to remove HNR_2_. The residue is redissolved by addition of ∼ 2 mL of equal amounts of anhydrous pyridine and acetonitrile. This is filtered to remove undissolved residue, and the filtrate is crystallized by storing the filtrate at −35 °C yielding colorless crystals of 2a, which were isolated by decanting the supernatant. Yield 0.050 g (68%).

##### Method B

21 mg (0.032 mmol) of Mn(NR_2_)_2_(THF)_2_ and 10 mg (0.056 mmol) of 3,5-diazido-1-amino-1,3,4-triazine were dissolved in THF. The reaction mixture stirred overnight and dried *in vacuo*. The residue was dissolved in ∼2 mL of equal amounts of anhydrous pyridine and acetonitrile and crystalized by slow evaporation under inert atmosphere. A single crystal was removed and identified by single-crystal X-ray diffraction. Unit cell: triclinic, *a* = 11.495(3) Å *b* = 11.697(3) Å *c* = 11.753(3)Å, *α* = 72.463(4)°, *β* = 82.359(4)°, *γ* = 71.422(3)° *V* = 1427.1(6) Å^3^. FTIR (cm^−1^): *ν* 2206, 2140 (N=C=N asymmetric stretch) 1545, 1351 (C–N, N–N stretches), 1245, 1211, 1071, 1038 (C–H bends), 841 (N=C=N bend), 753, 702 (Ph out-of-plane bend).

#### [(biTzI)Fe(Py)_3_]_2_ (2b)

0.022 g (0.034 mmol) of Fe(NR_2_)_2_(THF)_2_ is dissolved in pentane followed by the addition of 0.022 g (0.11 mmol) of cyanuric triazide giving a brown solution. This reaction mixture is stirred for 2.5 hours and the solvent is removed *in vacuo*. The dried product is dissolved in ∼2 mL of a 1 : 1 mixture of anhydrous pyridine and acetonitrile and kept at −35 °C from which colorless crystals of 2b were obtained in trace yield after two weeks at low temperature. Unit cell: triclinic, *a* = 11.034(4) Å *b* = 13.414(6) Å *c* = 18.758(8) Å, *α* = 91.221(6)°, *β* = 106.188(6)°, *γ* = 99.178(7)° *V* = 2625.8(19)Å^3^.

#### [(biTzI)Zn(Py)_3_]_2_ (2c)

0.04 g (0.061 mmol) of Zn(NR_2_)_2_ (0.10 mmol) is dissolved in anhydrous pentane followed by addition of 0.023 g (0.11 mmol) of cyanuric triazide to give an orange mixture. This reaction mixture is stirred at room temperature for three hours. The solvent is removed *in vacuo* and the product is dissolved in ∼2 mL 1 : 1 anhydrous pyridine and acetonitrile. Crystals of 2c are obtained after two days of evaporation at room temperature under anerobic atmosphere. Yield: 0.02 g, (40%). Unit cell: monoclinic P, *a* = 9.580(5) Å *b* = 13.076(8) Å *c* = 15.904(9) Å, *β* = 97.636(13)°, *V* = 1974.6(19) Å^3^. ^13^C NMR [ppm] (500 MHz, DMSO-d_6_): *δ*(ppm): 118.86 (N=C=N), 124.917, 137, 150 (Py), 154, 159 (Tz). FTIR (cm^−1^): *ν* 2198, 2064 (N=C=N asymmetric stretch), 1597, 1545 (C–N, N–N stretches), 1242, 1211 (C–H bends), 756, 696 (Ph out-of-plane bend).

#### [(biTzI)Cu(Py)_3_]_2_ (2d)

0.03 g (0.080 mmol) of tetrakis(acetonitrile)copper(i) hexafluorophosphate was dissolved in anhydrous tetrahydrofuran followed by the addition of 0.03 g (0.15 mmol) of cyanuric triazide which resulted in a dark green solution. This was stirred for two hours and dried *in vacuo*. The crude product is washed with anhydrous pentane and the residue is dissolved in ∼2 mL of 1 : 1 pyridine and acetonitrile, and filtered to remove undissolved residue. The filtrate is recrystallized by vapor diffusion with pentane over several days to give green crystals. Yield: 0.02 g (100% based on eqn (2)). Unit cell: monoclinic P, *a* = 9.332(3) Å *b* = 13.337(5) Å *c* = 15.845(6) Å, *β* = 97.844(7)°, *V* = 1953.6(12) Å^3^. FTIR (cm^−1^): *ν* 2204 (N=C=N asymmetric stretch), 1600, 1575, 1558 1542, 1351 (C–N, N–N stretch), 1257, 1214, 1211, 1090, 1071, 1042 (C–H bend), 838 (N=C=N bend), 762, 699, (Ph out-of-plane bend). UV-vis: nm (*ε*, M^−1^ cm^−1^), 700 (27 000).

#### [(biTzI)Ni(Py)_3_]_2_ (2e)

0.023 g (0.083 mmol) of Ni(COD)_2_ dissolved in anhydrous tetrahydrofuran followed by the addition of 0.023 g (0.11 mmol) of cyanuric azide giving a dark green solution. This reaction mixture is stirred for 2 hours and dried under vacuum. The dried product is washed with pentane and the residue is dissolved in ∼2 mL of 1 : 1 anhydrous pyridine and acetonitrile and filtered to remove undissolved residue. This solution is evaporated open to anaerobic atmosphere. Colorless crystals are obtained within 3–5 days. Yield: 0.007 g (18%). Unit cell: monoclinic P, *a* = 11.927(2) Å *b* = 20.144(4) Å *c* = 10.560(2) Å, *β* = 98.269(4)°, *V* = 2510.7(8) Å^3^.

#### 2-Amino-4,6-diazido-1,3,5triazine (3) from protonolysis of 2a

Compound 2a (0.05 g) is dissolved in 10 mL of 1 N HCl and stirred for one hour. Pentane is added and the organic product is precipitated at the interface between the layers. The liquid bilayer is carefully decanted and the solid is washed with pentane. The product is dried *in vacuo*, redissolved in methanol and recrystallized by slow evaporation which resulted in the formation of 3,5-diazido-1-amino-triazine. Weight obtained: 0.005 g, Yield: 26%. monoclinic P, *a* = 8.018(4) Å *b* = 4.669(3) Å *c* = 18.877(10) Å, *β* = 101.238(10)°, *V* = 693.1(7) Å^3^.

### Quantum chemical calculations

Electronic structure calculations were performed using the Gaussian 09,^68^ Molpro 2010,^69^ and ORCA 4.0 ^70^ program packages. The geometries of all structures corresponding to the stationary points on the potential energy surface (PES) of the species studied were fully optimized using density functional theory at the M06-2X/6-311++G(2df,p).^71^ All the equilibrium and transition state structures were ascertained to be minima and saddle points, respectively, on the potential energy surfaces. The nature of all localized transition states was verified using the intrinsic reaction coordinate (IRC) procedure. Zero-point energies and thermal corrections to enthalpy and Gibbs free energy were computed at the same DFT level of theory. Single-point electronic energies were afterward refined using the DLPNO-CCSD(T) methodology (the “NormalPNO” truncation thresholds were set)^55^ along with the aug-cc-pVQZ (aVQZ) basis set.^72^ The RIJK density fitting (DF) approximation^73^ was used to accelerate the convergence of the SCF components of DLPNO-CCSD(T) energy. The corresponding auxiliary basis sets (aug-cc-pVQZ/JK and aug-cc-pVQZ/C in the ORCA nomenclature)^71^ were used in the DF calculations of the SCF and correlation energies. In several cases, an explicitly correlated coupled-cluster formalism CCSD(T)-F12b was employed.^57^ Both DLPNO-CCSD(T) and CCSD(T)-F12 techniques have recently been shown to perform quite well for thermochemistry and kinetics of nitrogen-rich heterocycles.^74–77^ To account for the solvent effects, the free energies of solvation were calculated at the M06-2X/6-311++G(2df,p) level of theory using the polarized continuum model (PCM)^59^ for isotropic media with *ε* = 7.4 corresponding to tetrahydrofuran solution used in all syntheses. We used the gas-phase vibrational partition functions and single-point solvation energies for gas-phase optimized geometries, as was earlier benchmarked.^78^ The infrared spectrum of 2d (Fig. S9†) was calculated using the B3LYP/6-31G(d) level of theory.^79–81^

## Conclusions

In summary, a unique rearrangement of a six-membered aromatic polyazide 1 is reported that results from initial ligand reduction and elimination of N_2_ followed by cleavage of the six-membered triazine ring concomitant with formation of two new five-membered aromatic tetrazole rings. The initiating ligand reduction may be achieved *via* a ligand-based oxidation of trimethylsilylamido ligands to hydrazine, or by using reducing metal (Cu^I^ or Ni^0^) precursors. This product type appears to be stable and amenable at a number of transition metals, being isolable with at least five mid-late 3d transition metals and stabilizing them in their +2 oxidation states. Upon exposure to acid, the ligand product of the rearrangement undergoes a reverse reaction with the cleavage of the two five-membered azole rings regenerating the initial six-membered triazine ring. Future work will focus on the energetics of the acid/base dependent rearrangement, and the energetic properties of biTzI-type compounds.

## Conflicts of interest

There are no conflicts to declare.

## Supplementary Material

SC-012-D0SC04949B-s001

SC-012-D0SC04949B-s002
