# Supplementary material for: Transition-metal-mediated reduction and reversible double-cyclization of cyanuric triazide to an asymmetric bitetrazolate involving cleavage of the six-membered aromatic ring
Source: Chem Sci. 2020 Dec 8;12(6):2268–75. doi: 10.1039/d0sc04949b (PMC8179262; doi:10.1039/d0sc04949b)
Supplement: SC-012-D0SC04949B-s001 [file SC-012-D0SC04949B-s001.pdf]

Electronic Supplementary Material (ESI) for Chemical Science.
This journal is © The Royal Society of Chemistry 2020

SUPPORTING INFORMATION

Transition-metal-mediated reduction and reversible double-cyclization of cyanuric triazide to an asymmetric bitetrazolate involving cleavage of the six-membered aromatic ring

Shivaiah Vaddypally, Vitaly G. Kiselev, C. Alex N. Byrne, C. Franklin Goldsmith, Michael J. Zdilla

Contents

1. Thermal transformations of 1 and 1^- 4

2. NMR 5

3. IR spectra 7

4. UV-Visible Absorption Spectra 9

5. X-ray Crystallographic Tables....10

Table S1. Selected metal-ligand bond distances for compounds 2a – 2e.....10

2a....10

Table S2 Crystal data and structure refinement for 2a.....10

Table S3 Bond Lengths for 2a.....11

Table S4 Bond Angles for 2a.....12

Crystal structure determination of [2a] 12

Refinement model description 13

2b....14

Table S5 Crystal data and structure refinement for 2b. 14

Table S6 Bond Lengths for 2b.....15

Table S7 Bond Angles for 2b.....17

Crystal structure determination of [2b]....19

Refinement model description 19

2c....22

Table S8 Crystal data and structure refinement for 2c.....22

Table S9 Bond Lengths for 2c.....23

Table S10 Bond Angles for 2c. 24

Crystal structure determination of [2c] 24

Refinement model description 25

2d....26

Table S11 Crystal data and structure refinement for 2d. 26

Table S12 Bond Lengths for 2d.....27

Table S13 Bond Angles for 2d.....28

Crystal structure determination of [2d]....28

Refinement model description 29

2e....30

Table S14 Crystal data and structure refinement for 2e....30

Table S15 Bond Lengths for 2e. 31

Table S16 Bond Angles for 2e. 31
Crystal structure determination of [2e] 33
Refinement model description 33
3 37
Table S13 Crystal data and structure refinement for 3. 37
Table S14 Bond Lengths for 3. 38
Table S15 Bond Angles for 3. 38
Crystal structure determination of 3 38
Refinement model description 38
6. Raw quantum chemical data: 39
Reagent complex (nitrene+Zn) 39
TS1 40
I1 41
TS2 42
I2 43
TS3 44
P1 45

1. **Thermal transformations of 1 and 1⁻**

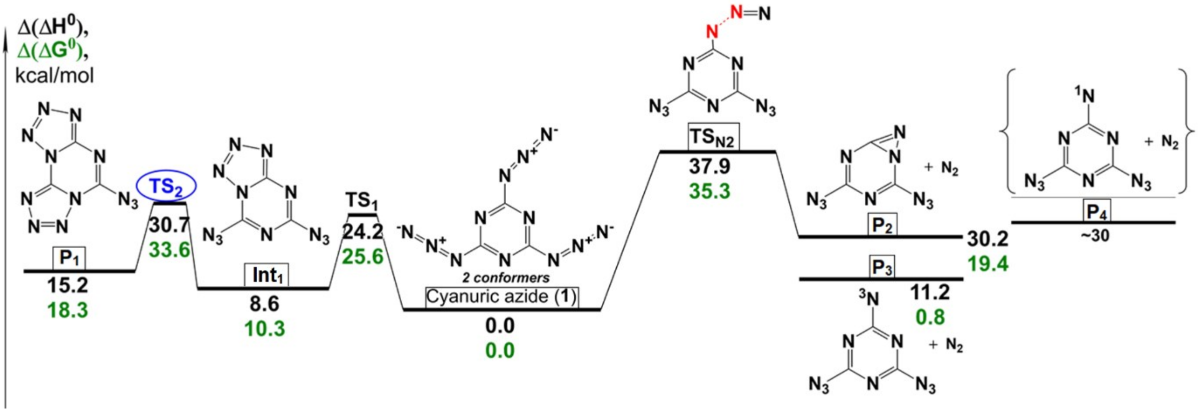

Figure S1. The stationary points on the PES of the most favorable thermal transormations of **1**. The relative thermodynamical potentials are calculated from the corresponding values for **1**. All values are calculated at the CCSD(T)-F12b/VDZ-F12 // M06-2X/6-311++G(2df,p) level of theory and are given in kcal/mol. Inset: relative enthalpies of the open-shell singlet nitrene estimated from the broken-symmetry calculations at the same DFT level of theory.

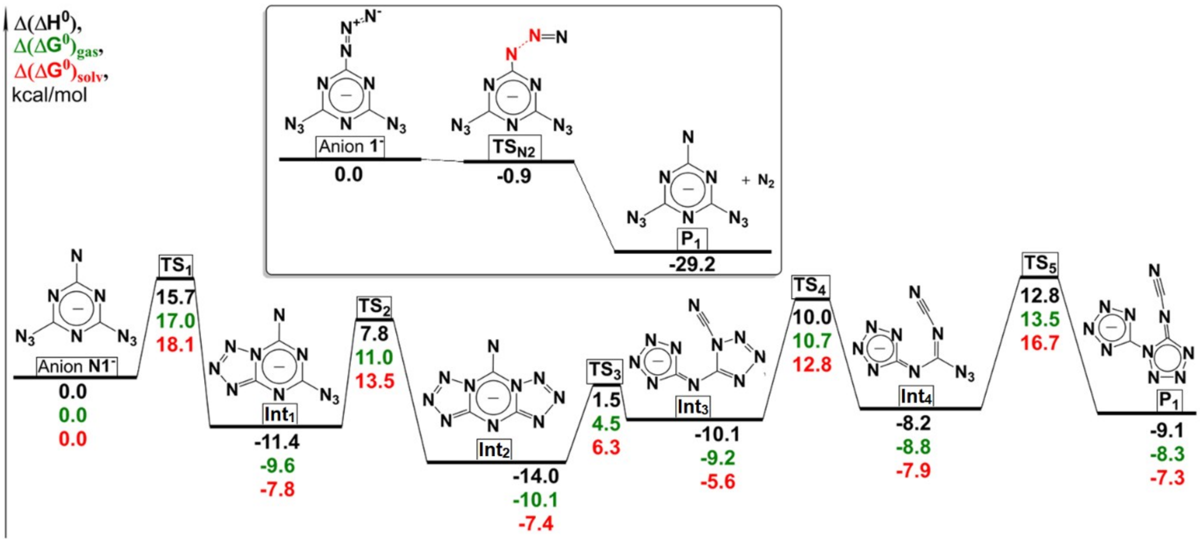

Figure S2. The stationary points on the PES of the most favorable thermal transformations of an anionic intermediate **N1⁻**. Inset: the N_2 elimination from the anion **1⁻**. The relative thermodynamical potentials are calculated from the corresponding values for **1⁻**. All values are calculated at the CCSD(T)-F12b/VDZ-F12 // M06-2X/6-311++G(2df,p) level of theory and are given in kcal/mol. The PCM free energies of solvation are calculated at the same DFT level of theory using tetrahydrofurane as a solvent. Regarding the N_2 elimination from the anion 1-. Note that the barrier of this reaction is close to zero, and the account of thermal corrections even leads to a slightly negative activation barrier.

2. NMR

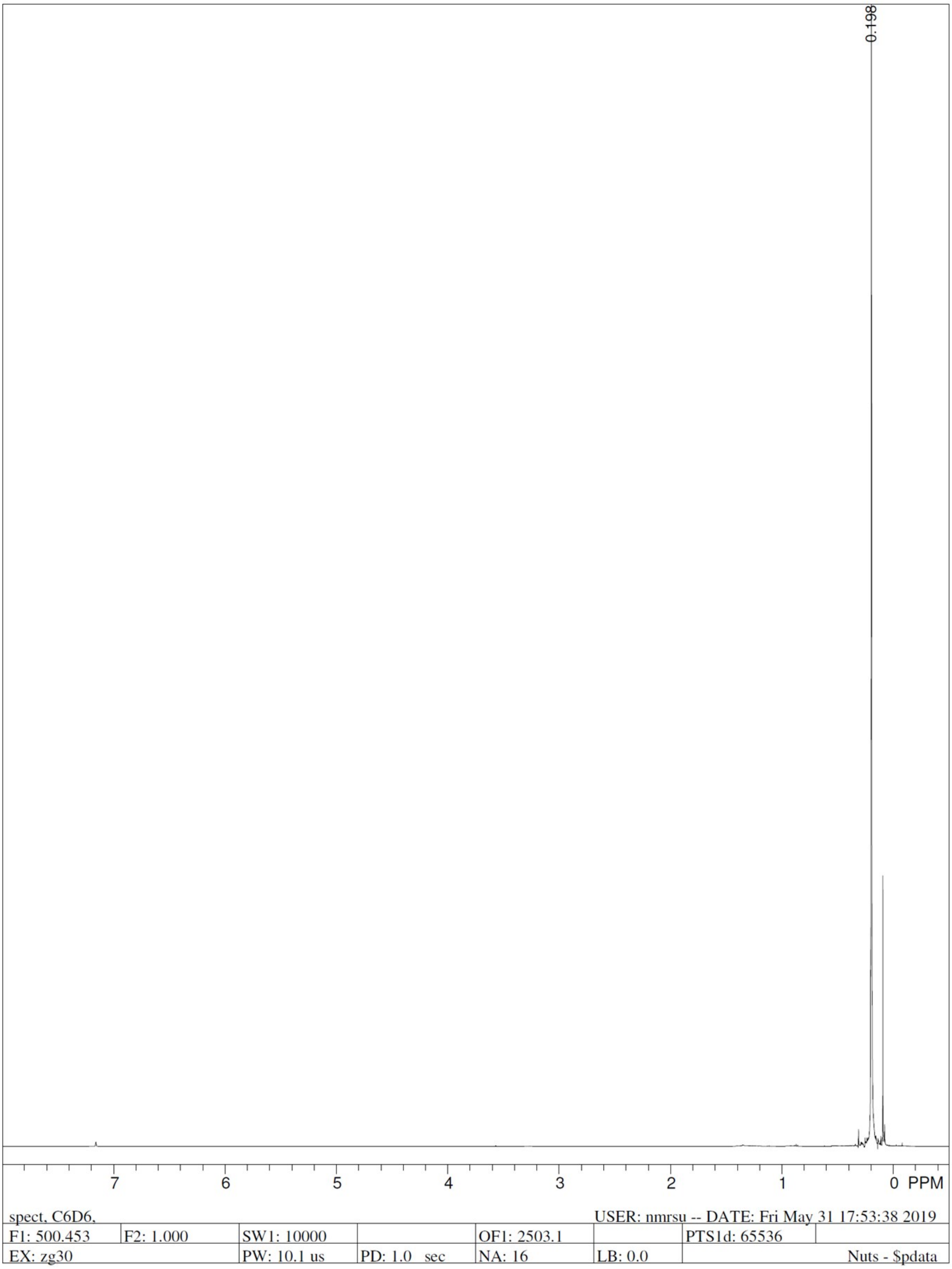

Figure S3. NMR spectrum of product tetrakis(trimethylsilyl)hydrazine at 0.2 ppm, as well as hexamethyldisilazane at 0.10 ppm in benzene-d_5.

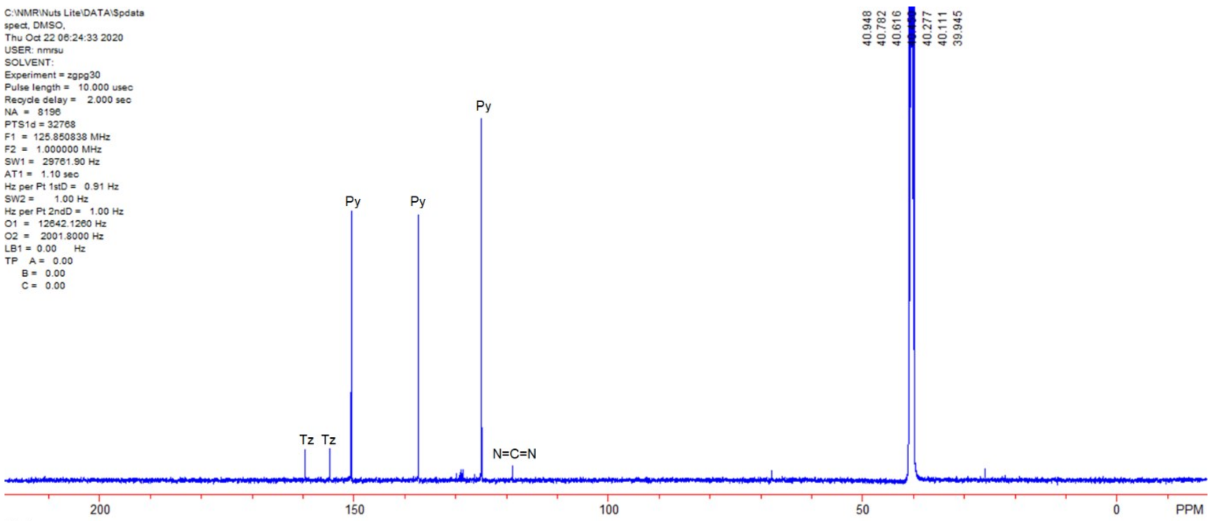

Figure S4. 500 MHz ^{13}C NMR of $[(biTzI)Zn(Py)_3]_2$ (**2c**) in DMSO. Residial protiosolvent signal shown at 40.45 ppm. Py, tetrazole (Tz), and carbodiimide (N=C=N) carbon signals assigned.

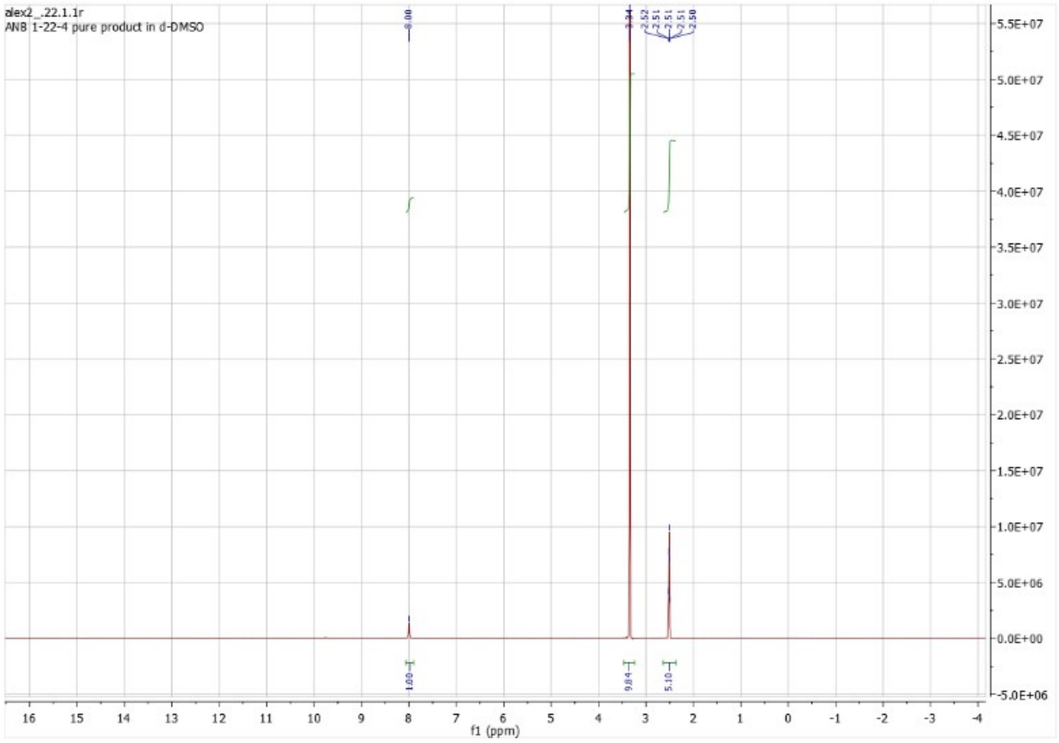

Figure S5. 500 MHz ^{1}H NMR spectrum of 2-amino-4,6-diazido-1,3,5 triazene in DMSO solvent. Residual solvent signal and contaminant water are apparent at 2.5 and 3.3 ppm respectively.

3. IR spectra

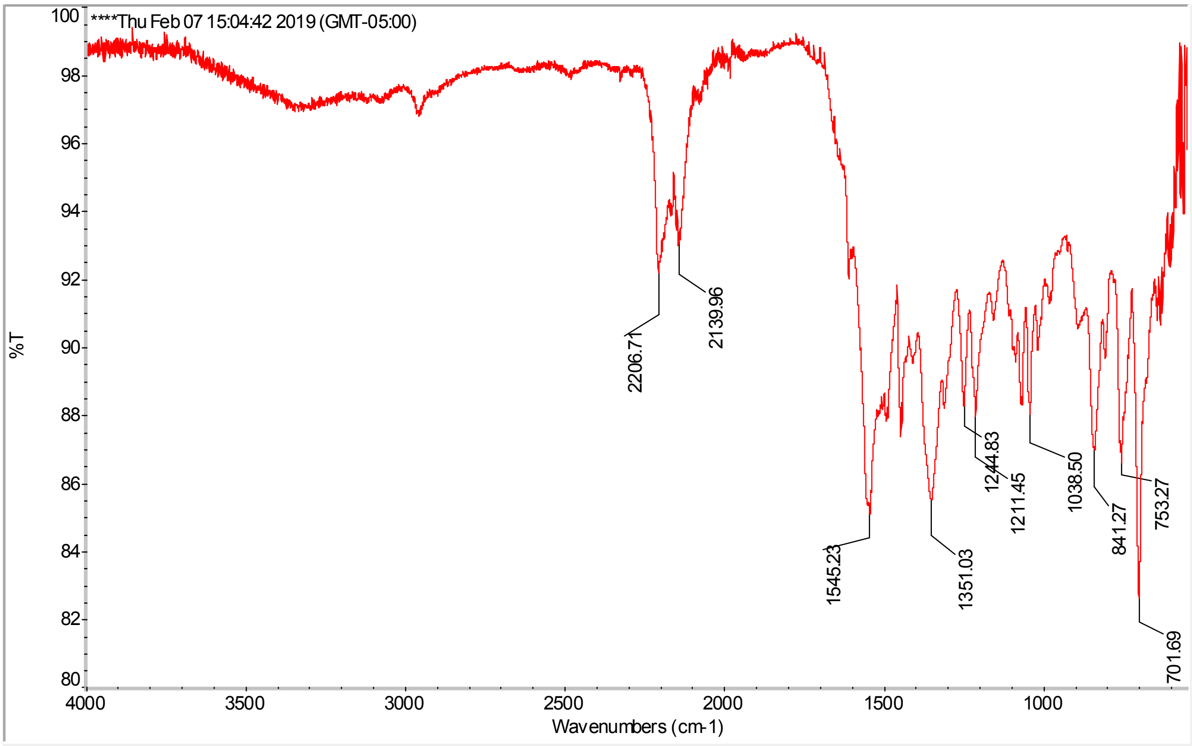

Figure S6. ATR-FTIR spectrum of **2a**.

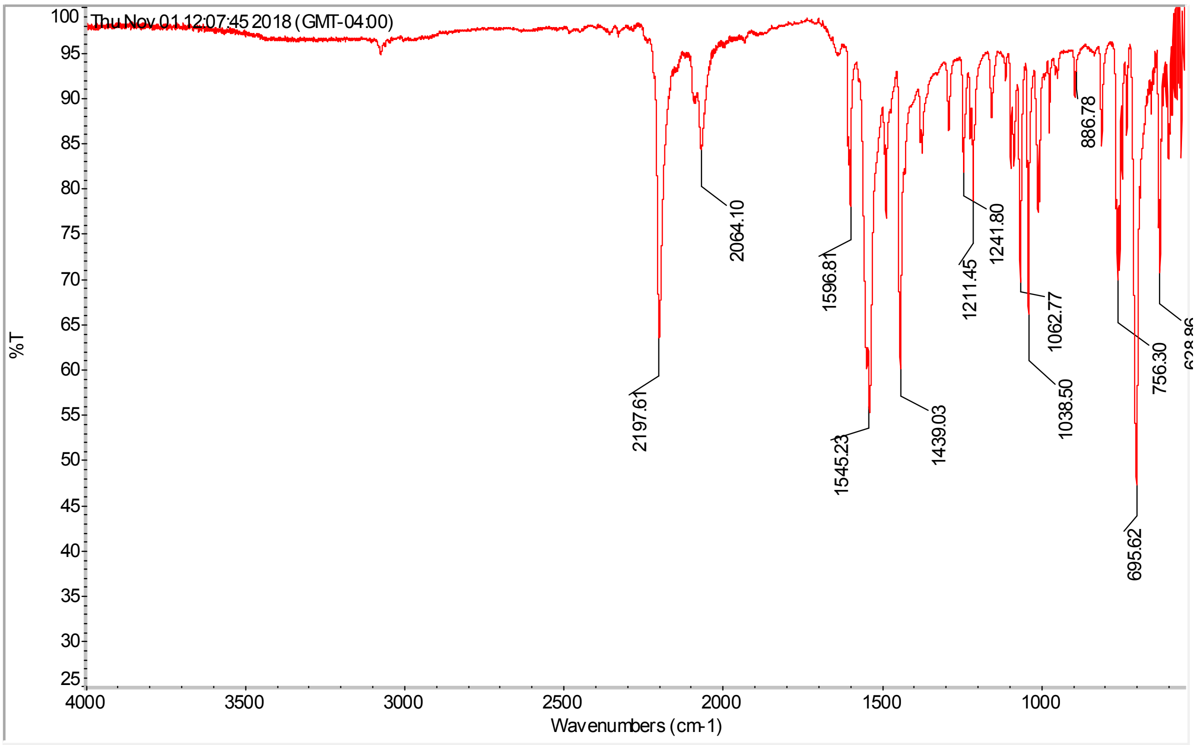

Figure S7. ATR-FTIR spectrum of **2c**.

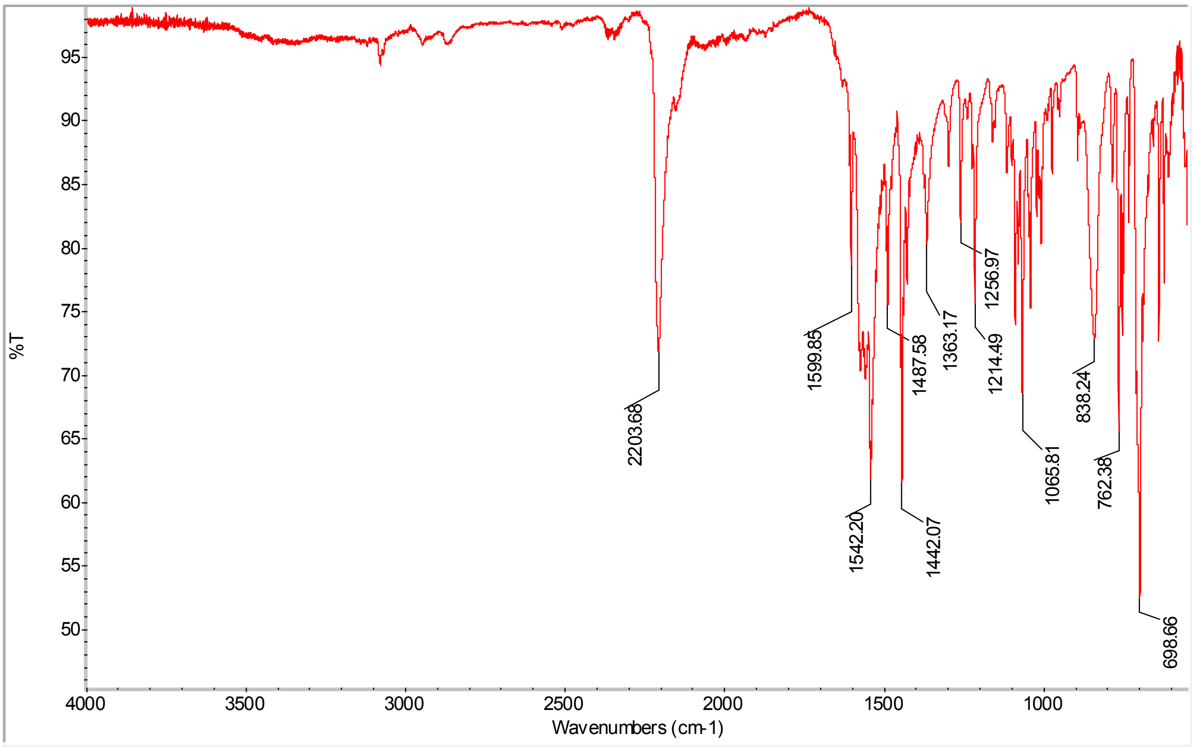

Figure S8. ATR-FTIR spectrum of **2d**.

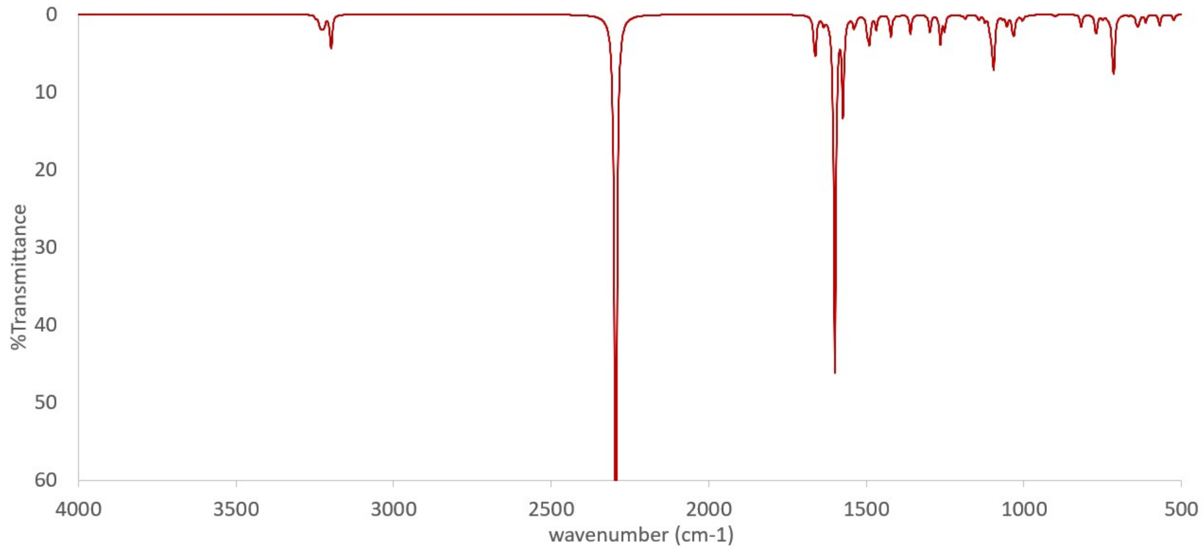

Figure S9. Computed IR spectrum using B3LYP/6-31G(d) level of theory.

4. UV-Visible Absorption Spectra

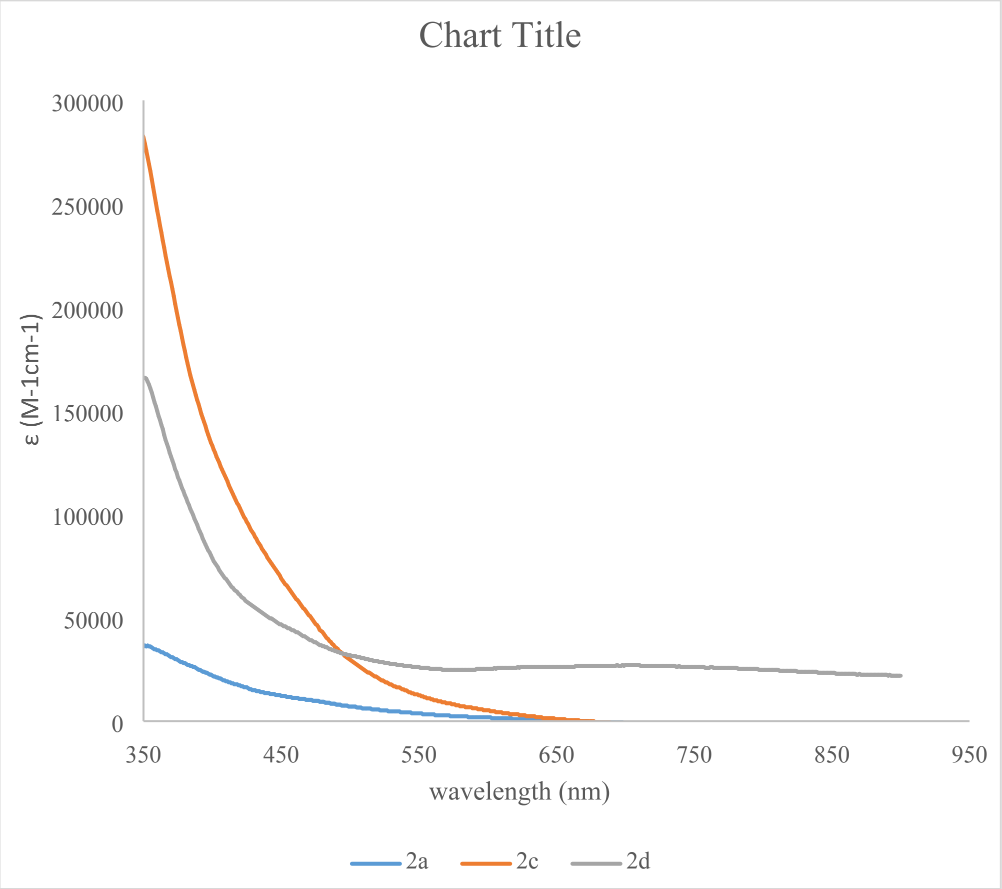

Figure S10. UV-Visible spectra of compounds 2a, 2c, 2d

5. X-ray Crystallographic Tables

Table S1. Selected metal-ligand bond distances for compounds 2a – 2e.[a]

	**1a** (M = Mn)	**1b** (M = Fe)	**1c** (M = Zn)	**1d** (M = Cu)	**1e** (M = Ni)
M(1)-N(1/2)	2.203(2)/2.3399(18)	2.122(6)/2.217(4)	2.105(5)/2.364(5)	1.971(4)/2.746(4)	2.087(2)/2.148(2)
M(1)-N(10/11)	2.221(2)/2.2335(19)	2.123(6)/2.163(4)	2.114(4)/2.144(5)	1.996(3)/2.278(3)	2.079(2)/2.085(2)
M(1)-N(21/31)	2.251(2)/2.301(2)	2.212(5)/2.178(5)	2.174(4)/2.217(5)	2.064(3)/2.070(3)	2.118(6)/2.097(2)
C1-N(1/2)	1.158(3)/1.304(3)	1.158(8)/1.291(8)	1.154(6)/1.306(7)	1.148(5)/1.299(5)	1.159(3)/1.309(3)

6. [a] Divided entries refer to separate, related atoms and their associated metrics in the order given, e.g., M(1)-N(1/2) denotes 2 distances: M(1)-N(1) and M(1)-N(2).

2a

Table S2 Crystal data and structure refinement for 2a.

Identification code	Mn
Empirical formula	$C_{50}H_{46}N_{30}Mn_2$
Formula weight	1177.05
Temperature/K	172.99
Crystal system	triclinic
Space group	P-1
a/Å	11.495(3)
b/Å	11.697(3)
c/Å	11.753(3)
α/°	72.463(4)
β/°	82.359(4)
γ/°	71.422(3)
Volume/Å^3	1427.2(6)
Z	1
ρ_{calc}g/cm^3	1.370
μ/mm^{-1}	0.507
F(000)	606.0
Crystal size/mm^3	0.17 × 0.05 × 0.024
Radiation	MoKα (λ = 0.71073)
2Θ range for data collection/°	3.638 to 55.906
Index ranges	$-15 \le h \le 14$, $-15 \le k \le 15$, $-15 \le l \le 15$
Reflections collected	11467
Independent reflections	6685 [R_{int} = 0.0173, R_{sigma} = 0.0286]
Data/restraints/parameters	6685/0/371
Goodness-of-fit on F^2	1.033
Final R indexes [I>=2σ (I)]	R_1 = 0.0500, wR_2 = 0.1281
Final R indexes [all data]	R_1 = 0.0644, wR_2 = 0.1410
Largest diff. peak/hole / e Å^{-3}	0.65/-0.60

Table S3 Bond Lengths for 2a.

Atom	Atom	Length/Å	Atom	Atom	Length/Å
Mn1	N1[1]	2.203(2)	N21	C26	1.337(3)
Mn1	N2	2.3399(18)	N31	C32	1.341(4)
Mn1	N10	2.221(2)	N31	C36	1.336(3)
Mn1	N11	2.2335(19)	C12	C13	1.384(4)
Mn1	N21	2.251(2)	C13	C14	1.367(5)
Mn1	N31	2.301(2)	C14	C15	1.367(5)
N1	C1	1.158(3)	C15	C16	1.381(4)
N2	C1	1.304(3)	C22	C23	1.384(4)
N2	C2	1.370(3)	C23	C24	1.369(5)
N3	N4	1.364(3)	C24	C25	1.379(4)
N3	C2	1.323(3)	C25	C26	1.381(4)
N4	N5	1.288(3)	C32	C33	1.376(5)
N5	N6	1.364(3)	C33	C34	1.380(6)
N6	C2	1.356(3)	C34	C35	1.372(5)
N6	C3	1.401(3)	C35	C36	1.375(4)
N10	N9	1.353(3)	N1S	C1S	1.131(7)
N10	C3	1.331(3)	C1S	C2S	1.448(8)
N9	N8	1.307(3)	N2S	C3S	1.262(7)
N8	N7	1.347(3)	N2S	C7S	1.297(8)
N7	C3	1.319(3)	C3S	C4S	1.275(8)
N11	C12	1.322(3)	C4S	C5S	1.258(9)
N11	C16	1.341(3)	C5S	C6S	1.328(10)
N21	C22	1.344(3)	C6S	C7S	1.375(9)

[1]2-X,2-Y,-Z

Table S4 Bond Angles for 2a.

Atom	Atom	Atom	Angle/°	Atom	Atom	Atom	Angle/°
N1[1]	Mn1	N2	88.94(7)	C26	N21	Mn1	119.56(15)
N1[1]	Mn1	N10	167.21(7)	C26	N21	C22	117.4(2)
N1[1]	Mn1	N11	95.45(7)	C32	N31	Mn1	119.5(2)
N1[1]	Mn1	N21	88.74(7)	C36	N31	Mn1	122.38(17)
N1[1]	Mn1	N31	88.91(8)	C36	N31	C32	117.4(2)
N10	Mn1	N2	78.52(7)	N1	C1	N2	173.6(2)
N10	Mn1	N11	96.92(7)	N3	C2	N2	129.6(2)
N10	Mn1	N21	93.62(7)	N3	C2	N6	108.52(19)
N10	Mn1	N31	87.52(8)	N6	C2	N2	121.85(19)
N11	Mn1	N2	174.46(7)	N10	C3	N6	122.1(2)
N11	Mn1	N21	94.13(8)	N7	C3	N6	124.2(2)
N11	Mn1	N31	91.39(8)	N7	C3	N10	113.6(2)
N21	Mn1	N2	89.32(7)	N11	C12	C13	122.9(3)
N21	Mn1	N31	174.18(7)	C14	C13	C12	119.0(3)
N31	Mn1	N2	85.31(7)	C13	C14	C15	118.9(3)
C1	N1	Mn1[1]	146.83(18)	C14	C15	C16	119.0(3)
C1	N2	Mn1	118.75(13)	N11	C16	C15	122.6(3)
C1	N2	C2	117.09(17)	N21	C22	C23	122.7(3)
C2	N2	Mn1	117.92(13)	C24	C23	C22	119.2(3)
C2	N3	N4	105.3(2)	C23	C24	C25	118.8(3)
N5	N4	N3	112.4(2)	C24	C25	C26	118.9(3)
N4	N5	N6	105.6(2)	N21	C26	C25	123.1(2)
N5	N6	C3	122.4(2)	N31	C32	C33	123.3(3)
C2	N6	N5	108.26(19)	C32	C33	C34	118.3(3)
C2	N6	C3	129.17(19)	C35	C34	C33	118.9(3)
N9	N10	Mn1	129.21(15)	C34	C35	C36	119.3(3)
C3	N10	Mn1	127.15(16)	N31	C36	C35	122.7(3)
C3	N10	N9	103.56(19)	N1S	C1S	C2S	179.3(6)
N8	N9	N10	109.0(2)	C3S	N2S	C7S	120.6(6)
N9	N8	N7	110.4(2)	N2S	C3S	C4S	123.6(7)
C3	N7	N8	103.4(2)	C5S	C4S	C3S	121.1(7)
C12	N11	Mn1	121.45(17)	C4S	C5S	C6S	117.9(6)
C12	N11	C16	117.7(2)	C5S	C6S	C7S	121.2(6)
C16	N11	Mn1	120.82(17)	N2S	C7S	C6S	115.6(6)
C22	N21	Mn1	122.88(18)				

[1]2-X,2-Y,-Z

Crystal structure determination of [2a]

Crystal Data for $C_{50}H_{46}N_{30}Mn_2$ (M =1177.05 g/mol): triclinic, space group P-1 (no. 2), a = 11.495(3) Å, b = 11.697(3) Å, c = 11.753(3) Å, α = 72.463(4)°, β = 82.359(4)°, γ = 71.422(3)°, V = 1427.2(6) Å^3, Z = 1, T = 172.99 K, μ(MoKα) = 0.507 mm^{-1}, $Dcalc$ = 1.370 g/cm^3, 11467 reflections measured (3.638° ≤ 2Θ ≤ 55.906°), 6685 unique (R_{int} = 0.0173, R_{sigma} = 0.0286) which were used in all calculations. The final R_1 was 0.0500 (I > 2σ(I)) and wR_2 was 0.1410 (all data).

Refinement model description

Number of restraints - 0

Details: No disorder modelling was necessary. Hydrogen atoms were refined as riding models.

1. Fixed Uiso
 At 1.2 times of:
  All C(H) groups
 At 1.5 times of:
  All C(H,H,H) groups
2.a Aromatic/amide H refined with riding coordinates:
 C12(H12), C13(H13), C14(H14), C15(H15), C16(H16), C22(H22), C23(H23), C24(H24), C25(H25), C26(H26), C32(H32), C33(H33), C34(H34), C35(H35), C36(H36), C3S(H3S), C4S(H4S), C5S(H5S), C6S(H6S), C7S(H7S)
2.b Idealised Me refined as rotating group:
 C2S(H2SA,H2SB,H2SC)

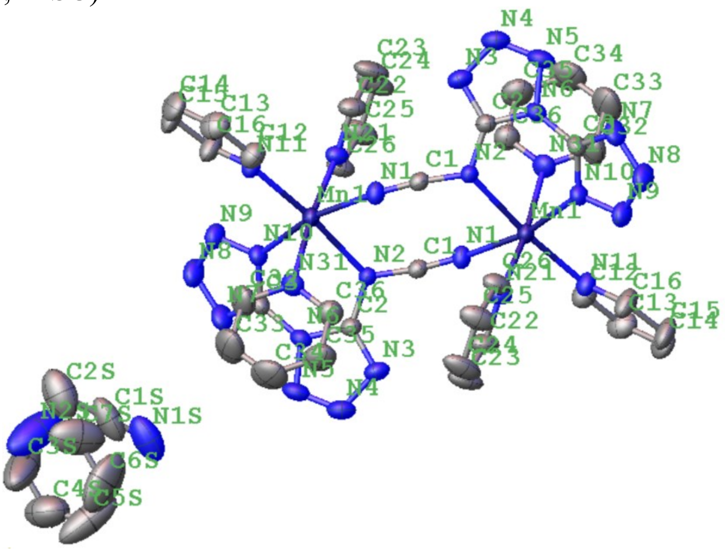

Figure S11. Thermal Ellipsoid plot of **2a**·Py·MeCN. Ellipsoids set at 50% probability. Hydrogen atoms omitted for clarity.

2b

Table S5 Crystal data and structure refinement for 2b.

Empirical formula	$C_{46}H_{49}N_{27}Fe_2O_2$
Formula weight	1123.82
Temperature/K	100.04
Crystal system	triclinic
Space group	P-1
a/Å	11.034(4)
b/Å	13.414(6)
c/Å	18.758(8)
α/°	91.221(6)
β/°	106.188(6)
γ/°	99.178(7)
Volume/Å^3	2625.7(19)
Z	2
ρ_{calc}g/cm^3	1.421
μ/mm^{-1}	0.620
F(000)	1164.0
Crystal size/mm^3	0.15 × 0.096 × 0.037
Radiation	MoKα (λ = 0.71073)
2Θ range for data collection/°	2.266 to 49.418
Index ranges	$-6 \leq h \leq 12$, $-15 \leq k \leq 15$, $-22 \leq l \leq 22$
Reflections collected	14524
Independent reflections	8515 [R_{int} = 0.0496, R_{sigma} = 0.1020]
Data/restraints/parameters	8515/590/842
Goodness-of-fit on F^2	1.014
Final R indexes [I>=2σ (I)]	R_1 = 0.0685, wR_2 = 0.1671
Final R indexes [all data]	R_1 = 0.1470, wR_2 = 0.2186
Largest diff. peak/hole / e Å^{-3}	1.25/-0.79

Table S6 Bond Lengths for 2b.

Atom	Atom	Length/Å	Atom	Atom	Length/Å
Fe1	N1[1]	2.122(6)	N46	C43	1.405(8)
Fe1	N2	2.217(4)	N47	N48	1.357(8)
Fe1	N10	2.123(6)	N47	C43	1.298(9)
Fe1	N11	2.163(4)	N48	N49	1.284(9)
Fe1	N21	2.212(5)	N49	N50	1.347(7)
Fe1	N31	2.178(5)	N50	C43	1.316(8)
N1	C1	1.158(8)	N51	C52	1.332(13)
N2	C1	1.291(8)	N51	C56	1.297(13)
N2	C2	1.350(7)	N51A	C52A	1.342(15)
N3	N4	1.372(7)	N51A	C56A	1.302(15)
N3	C2	1.317(7)	C52A	C53A	1.399(15)
N4	N5	1.280(7)	C53A	C54A	1.338(19)
N5	N6	1.349(7)	C54A	C55A	1.370(18)
N6	C2	1.342(8)	C55A	C56A	1.366(14)
N6	C3	1.405(7)	N61	C62	1.342(8)
N7	N8	1.362(6)	N61	C66	1.333(8)
N7	C3	1.300(8)	N71	C72	1.333(9)
N8	N9	1.291(7)	N71	C76	1.337(8)
N9	N10	1.352(7)	C52	C53	1.396(13)
N10	C3	1.317(7)	C53	C54	1.357(17)
N11	C12	1.355(9)	C54	C55	1.358(16)
N11	C16	1.326(9)	C55	C56	1.371(13)
N21	C22	1.348(8)	C62	C63	1.365(9)
N21	C26	1.326(8)	C63	C64	1.368(10)
N31	C32	1.322(8)	C64	C65	1.355(10)
N31	C36	1.343(8)	C65	C66	1.359(9)
C12	C13	1.384(8)	C72	C73	1.348(10)
C13	C14	1.384(10)	C73	C74	1.360(11)
C14	C15	1.357(10)	C74	C75	1.366(12)
C15	C16	1.379(8)	C75	C76	1.347(10)
C22	C23	1.351(9)	O1S	C3S	1.422(8)
C23	C24	1.365(11)	O1S	C6S	1.432(9)
C24	C25	1.380(11)	C3S	C4S	1.537(9)
C25	C26	1.359(10)	C4S	C5S	1.526(9)
C32	C33	1.354(9)	C5S	C6S	1.563(9)
C33	C34	1.376(10)	O1T	C3T	1.451(10)
C34	C35	1.361(9)	O1T	C6T	1.440(10)
C35	C36	1.366(9)	C3T	C4T	1.550(10)
Fe2	N41[2]	2.125(6)	C4T	C5T	1.559(10)
Fe2	N42	2.211(5)	C5T	C6T	1.548(10)
Fe2	N50	2.137(6)	O2S	C7S	1.454(10)
Fe2	N51	2.146(16)	O2S	C10S	1.456(10)
Fe2	N51A	2.21(2)	C7S	C8S	1.550(10)

Atom	Atom	Length/Å	Atom	Atom	Length/Å
Fe2	N61	2.185(5)	C8S	C9S	1.558(10)
Fe2	N71	2.164(5)	C9S	C10S	1.558(10)
N41	C41	1.140(8)	O2T	C7T	1.422(9)
N42	C41	1.313(8)	O2T	C10T	1.449(10)
N42	C42	1.352(8)	C7T	C8T	1.524(10)
N43	N44	1.361(8)	C8T	C9T	1.564(9)
N43	C42	1.322(7)	C9T	C10T	1.549(10)
N44	N45	1.290(8)	N1S	C1S	1.125(10)
N45	N46	1.357(7)	C1S	C2S	1.446(12)
N46	C42	1.331(8)			

[1]2-X,1-Y,2-Z; [2]-X,2-Y,1-Z

Table S7 Bond Angles for 2b.

Atom	Atom	Atom	Angle/°	Atom	Atom	Atom	Angle/°
N1[1]	Fe1	N2	89.74(18)	N71	Fe2	N42	89.95(19)
N1[1]	Fe1	N10	171.78(17)	N71	Fe2	N51A	93.9(10)
N1[1]	Fe1	N11	94.52(19)	N71	Fe2	N61	176.99(19)
N1[1]	Fe1	N21	88.8(2)	C41	N41	Fe2[2]	148.4(5)
N1[1]	Fe1	N31	88.3(2)	C41	N42	Fe2	119.6(4)
N10	Fe1	N2	82.25(18)	C41	N42	C42	116.7(5)
N10	Fe1	N11	93.46(19)	C42	N42	Fe2	118.9(4)
N10	Fe1	N21	88.9(2)	C42	N43	N44	105.2(5)
N10	Fe1	N31	93.4(2)	N45	N44	N43	111.9(5)
N11	Fe1	N2	175.6(2)	N44	N45	N46	105.2(5)
N11	Fe1	N21	92.12(19)	N45	N46	C43	122.4(6)
N11	Fe1	N31	92.27(19)	C42	N46	N45	109.2(5)
N21	Fe1	N2	87.02(18)	C42	N46	C43	128.3(5)
N31	Fe1	N2	88.79(18)	C43	N47	N48	102.2(6)
N31	Fe1	N21	174.91(18)	N49	N48	N47	110.8(6)
C1	N1	Fe1[1]	147.7(5)	N48	N49	N50	108.9(6)
C1	N2	Fe1	118.9(4)	N49	N50	Fe2	130.7(4)
C1	N2	C2	117.2(5)	C43	N50	Fe2	126.0(4)
C2	N2	Fe1	119.1(3)	C43	N50	N49	103.3(6)
C2	N3	N4	105.1(5)	C52	N51	Fe2	121.8(11)
N5	N4	N3	111.7(5)	C56	N51	Fe2	120.7(11)
N4	N5	N6	106.0(5)	C56	N51	C52	117.4(11)
N5	N6	C3	122.1(5)	C52A	N51A	Fe2	119.4(14)
C2	N6	N5	108.8(4)	C56A	N51A	Fe2	122.3(14)
C2	N6	C3	129.0(5)	C56A	N51A	C52A	117.5(14)
C3	N7	N8	102.7(5)	N51A	C52A	C53A	121.8(16)
N9	N8	N7	110.2(5)	C54A	C53A	C52A	118.3(15)
N8	N9	N10	109.1(5)	C53A	C54A	C55A	120.1(14)
N9	N10	Fe1	129.8(4)	C56A	C55A	C54A	117.9(15)
C3	N10	Fe1	126.8(4)	N51A	C56A	C55A	124.2(15)
C3	N10	N9	103.4(5)	C62	N61	Fe2	120.6(5)
C12	N11	Fe1	120.4(4)	C66	N61	Fe2	121.8(4)
C16	N11	Fe1	121.6(4)	C66	N61	C62	117.4(6)
C16	N11	C12	118.0(5)	C72	N71	Fe2	121.6(4)
C22	N21	Fe1	120.5(4)	C72	N71	C76	115.8(6)
C26	N21	Fe1	122.6(4)	C76	N71	Fe2	122.2(5)
C26	N21	C22	116.3(6)	N41	C41	N42	173.7(6)
C32	N31	Fe1	122.9(5)	N43	C42	N42	129.5(6)
C32	N31	C36	117.5(6)	N43	C42	N46	108.5(6)
C36	N31	Fe1	119.5(4)	N46	C42	N42	121.9(5)
N1	C1	N2	173.6(6)	N47	C43	N46	123.0(6)
N3	C2	N2	129.4(6)	N47	C43	N50	114.8(6)
N3	C2	N6	108.5(5)	N50	C43	N46	122.2(6)

Atom	Atom	Atom	Angle/°	Atom	Atom	Atom	Angle/°
N6	C2	N2	122.1(5)	N51	C52	C53	122.4(13)
N7	C3	N6	123.3(5)	C54	C53	C52	118.3(12)
N7	C3	N10	114.7(5)	C53	C54	C55	119.0(11)
N10	C3	N6	122.0(6)	C54	C55	C56	118.9(13)
N11	C12	C13	121.9(7)	N51	C56	C55	123.9(13)
C14	C13	C12	118.2(7)	N61	C62	C63	121.5(7)
C15	C14	C13	120.2(6)	C62	C63	C64	119.9(7)
C14	C15	C16	118.4(7)	C65	C64	C63	118.9(7)
N11	C16	C15	123.3(7)	C64	C65	C66	118.6(7)
N21	C22	C23	123.4(7)	N61	C66	C65	123.6(6)
C22	C23	C24	119.4(7)	N71	C72	C73	122.9(7)
C23	C24	C25	118.3(7)	C72	C73	C74	120.1(8)
C26	C25	C24	118.7(7)	C73	C74	C75	118.4(8)
N21	C26	C25	123.9(7)	C76	C75	C74	118.0(8)
N31	C32	C33	123.6(7)	N71	C76	C75	124.8(8)
C32	C33	C34	118.8(7)	C3S	O1S	C6S	108.8(7)
C35	C34	C33	118.4(7)	O1S	C3S	C4S	111.7(8)
C34	C35	C36	119.7(7)	C5S	C4S	C3S	103.2(7)
N31	C36	C35	121.9(6)	C4S	C5S	C6S	105.2(8)
N41[2]	Fe2	N42	87.91(18)	O1S	C6S	C5S	108.6(7)
N41[2]	Fe2	N50	169.44(18)	C6T	O1T	C3T	108.2(11)
N41[2]	Fe2	N51	94.0(7)	O1T	C3T	C4T	109.3(12)
N41[2]	Fe2	N51A	87.0(8)	C3T	C4T	C5T	104.0(10)
N41[2]	Fe2	N61	89.1(2)	C6T	C5T	C4T	104.6(9)
N41[2]	Fe2	N71	88.5(2)	O1T	C6T	C5T	110.5(10)
N50	Fe2	N42	81.75(19)	C7S	O2S	C10S	105.4(14)
N50	Fe2	N51	96.3(7)	O2S	C7S	C8S	107.7(13)
N50	Fe2	N51A	103.1(8)	C7S	C8S	C9S	104.8(9)
N50	Fe2	N61	88.5(2)	C10S	C9S	C8S	103.2(11)
N50	Fe2	N71	93.6(2)	O2S	C10S	C9S	106.8(14)
N51	Fe2	N42	177.9(8)	C7T	O2T	C10T	108.6(9)
N51	Fe2	N61	91.2(7)	O2T	C7T	C8T	108.5(9)
N51	Fe2	N71	90.8(8)	C7T	C8T	C9T	102.3(9)
N51A	Fe2	N42	173.6(7)	C10T	C9T	C8T	102.0(8)
N61	Fe2	N42	88.17(18)	O2T	C10T	C9T	108.9(9)
N61	Fe2	N51A	87.8(10)	N1S	C1S	C2S	175.3(11)

[1]2-X,1-Y,2-Z; [2]-X,2-Y,1-Z

Crystal structure determination of [2b]

Crystal Data for $C_{46}H_{49}N_{27}Fe_2O_2$ (M =1123.82 g/mol): triclinic, space group P-1 (no. 2), a = 11.034(4) Å, b = 13.414(6) Å, c = 18.758(8) Å, α = 91.221(6)°, β = 106.188(6)°, γ = 99.178(7)°, V = 2625.7(19) Å^3, Z = 2, T = 100.04 K, μ(MoKα) = 0.620 mm^{-1}, $Dcalc$ = 1.421 g/cm^3, 14524 reflections measured (2.266° ≤ 2Θ ≤ 49.418°), 8515 unique (R_{int} = 0.0496, R_{sigma} = 0.1020) which were used in all calculations. The final R_1 was 0.0685 (I > 2σ(I)) and wR_2 was 0.2186 (all data).

Refinement model description

Number of restraints - 590.

Details: Lattice THF molecules were disordered. Split atom models were used along with distance and thermal restraints to deal with parameter correlation. All hydrogen atoms refined as riding models. The largest residual peak with electron count of 1.2 was located near Fe, and was less than 10% of the atomic number of iron (26).

1. Fixed Uiso
At 1.2 times of:
All C(H) groups, All C(H,H) groups
At 1.5 times of:
All C(H,H,H) groups
2. Restrained distances
O2S-C10S = O2S-C7S = O1S-C3S = O1S-C6S
1.45 with sigma of 0.01
O2T-C10T = O2T-C7T = O1T-C3T = O1T-C6T
1.45 with sigma of 0.01
C9S-C10S = C9S-C8S = C8S-C7S = C4S-C3S = C5S-C4S = C6S-C5S
1.54 with sigma of 0.01
C9T-C10T = C9T-C8T = C8T-C7T = C4T-C3T = C5T-C4T = C6T-C5T
1.54 with sigma of 0.01
C7S-C10S
2.29 with sigma of 0.02
C7T-C10T
2.29 with sigma of 0.02
C6S-C3S
2.29 with sigma of 0.02
C6T-C3T
2.29 with sigma of 0.02
O2S-C8S
2.44 with sigma of 0.02
O2T-C8T
2.44 with sigma of 0.02
O2S-C9S
2.44 with sigma of 0.02
O2T-C9T
2.44 with sigma of 0.02
O1S-C5S
2.44 with sigma of 0.02
O1T-C5T
2.44 with sigma of 0.02
O1S-C4S
2.44 with sigma of 0.02
O1T-C4T

2.44 with sigma of 0.02
C10S-C8S
2.52 with sigma of 0.02
C10T-C8T
2.52 with sigma of 0.02
C9S-C7S
2.52 with sigma of 0.02
C9T-C7T
2.52 with sigma of 0.02
C3S-C5S
2.52 with sigma of 0.02
C3T-C5T
2.52 with sigma of 0.02
C6S-C4S
2.52 with sigma of 0.02
C6T-C4T
2.52 with sigma of 0.02

3. Rigid bond restraints
N51, N51A, C52, C52A, C53, C53A, C54, C54A, C55, C55A, C56, C56A
with sigma for 1-2 distances of 0.01 and sigma for 1-3 distances of 0.01
O2S, C7S, C8S, C9S, C10S, O2T, C7T, C8T, C9T, C10T
with sigma for 1-2 distances of 0.03 and sigma for 1-3 distances of 0.03
O1S, C3S, C4S, C5S, C6S, O1T, C3T, C4T, C5T, C6T
with sigma for 1-2 distances of 0.03 and sigma for 1-3 distances of 0.03

4. Uiso/Uaniso restraints and constraints
N51 ≈ N51A ≈ C52 ≈ C52A ≈ C53 ≈ C53A ≈ C54 ≈ C54A
≈ C55 ≈ C55A ≈ C56 ≈ C56A: within 2A with sigma of 0.01 and
sigma for terminal atoms of 0.02
O2S ≈ C7S ≈ C8S ≈ C9S ≈ C10S ≈ O2T ≈ C7T ≈ C8T
≈ C9T ≈ C10T: within 2A with sigma of 0.03 and sigma for terminal atoms
of 0.06
O1S ≈ C3S ≈ C4S ≈ C5S ≈ C6S ≈ O1T ≈ C3T ≈ C4T ≈
C5T ≈ C6T: within 2A with sigma of 0.03 and sigma for terminal atoms of 0.06

5. Same fragment restrains
[N51, C52, C53, C54, C55, C56] sigma for 1-2: 0.02, 1-3: 0.04
as
[N51A, C52A, C53A, C54A, C55A, C56A]

6. Others
Sof(N51A)=Sof(C52A)=Sof(H52A)=Sof(C53A)=Sof(H53A)=Sof(C54A)=Sof(H54A)=
Sof(C55A)=Sof(H55A)=Sof(C56A)=Sof(H56A)=1-FVAR(1)
Sof(N51)=Sof(C52)=Sof(H52)=Sof(C53)=Sof(H53)=Sof(C54)=Sof(H54)=Sof(C55)=
Sof(H55)=Sof(C56)=Sof(H56)=FVAR(1)

Sof(O2T)=Sof(C7T)=Sof(H7TA)=Sof(H7TB)=Sof(C8T)=Sof(H8TA)=Sof(H8TB)=Sof(C9T
)=
Sof(H9TA)=Sof(H9TB)=Sof(C10T)=Sof(H10C)=Sof(H10D)=1-FVAR(2)

Sof(O2S)=Sof(C7S)=Sof(H7SA)=Sof(H7SB)=Sof(C8S)=Sof(H8SA)=Sof(H8SB)=Sof(C9S)
=
Sof(H9SA)=Sof(H9SB)=Sof(C10S)=Sof(H10A)=Sof(H10B)=FVAR(2)

Sof(O1T)=Sof(C3T)=Sof(H3TA)=Sof(H3TB)=Sof(C4T)=Sof(H4TA)=Sof(H4TB)=Sof(C5T
)=
 Sof(H5TA)=Sof(H5TB)=Sof(C6T)=Sof(H6TA)=Sof(H6TB)=1-FVAR(3)

Sof(O1S)=Sof(C3S)=Sof(H3SA)=Sof(H3SB)=Sof(C4S)=Sof(H4SA)=Sof(H4SB)=Sof(C5S)
=
 Sof(H5SA)=Sof(H5SB)=Sof(C6S)=Sof(H6SA)=Sof(H6SB)=FVAR(3)

7.a Secondary CH2 refined with riding coordinates:
 C3S(H3SA,H3SB), C4S(H4SA,H4SB), C5S(H5SA,H5SB), C6S(H6SA,H6SB), C3T(H3TA,
 H3TB), C4T(H4TA,H4TB), C5T(H5TA,H5TB), C6T(H6TA,H6TB), C7S(H7SA,H7SB),
 C8S(H8SA,H8SB), C9S(H9SA,H9SB), C10S(H10A,H10B), C7T(H7TA,H7TB),
C8T(H8TA,
 H8TB), C9T(H9TA,H9TB), C10T(H10C,H10D)

7.b Aromatic/amide H refined with riding coordinates:
 C12(H12), C13(H13), C14(H14), C15(H15), C16(H16), C22(H22), C23(H23),
 C24(H24), C25(H25), C26(H26), C32(H32), C33(H33), C34(H34), C35(H35), C36(H36),
 C52A(H52A), C53A(H53A), C54A(H54A), C55A(H55A), C56A(H56A), C52(H52),
 C53(H53), C54(H54), C55(H55), C56(H56), C62(H62), C63(H63), C64(H64), C65(H65),
 C66(H66), C72(H72), C73(H73), C74(H74), C75(H75), C76(H76)

7.c Idealised Me refined as rotating group:
 C2S(H2SA,H2SB,H2SC)

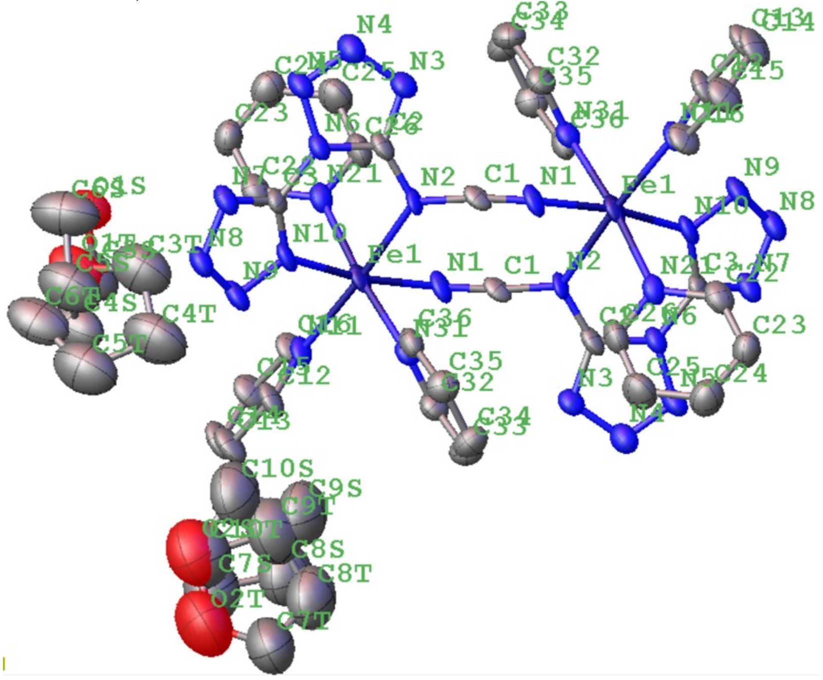

Figure S12. Thermal Ellipsoid plot of **2b**. Ellipsoids set at 50% probability. Hydrogen atoms omitted for clarity.

2c

Table S8 Crystal data and structure refinement for 2c.

Identification code	2c
Empirical formula	$C_{36}H_{30}N_{26}Zn_2$
Formula weight	957.60
Temperature/K	100.03
Crystal system	monoclinic
Space group	$P2_1/n$
a/Å	9.580(5)
b/Å	13.076(8)
c/Å	15.904(9)
α/°	90
β/°	97.636(13)
γ/°	90
Volume/Å^3	1974.5(19)
Z	2
ρ_{calc}g/cm^3	1.611
μ/mm^{-1}	1.283
F(000)	976.0
Crystal size/mm^3	0.15 × 0.08 × 0.03
Radiation	MoKα (λ = 0.71073)
2Θ range for data collection/°	4.048 to 49.414
Index ranges	$-11 \le h \le 9$, $-13 \le k \le 15$, $-15 \le l \le 18$
Reflections collected	8344
Independent reflections	3376 [R_{int} = 0.0873, R_{sigma} = 0.1280]
Data/restraints/parameters	3376/0/289
Goodness-of-fit on F^2	0.988
Final R indexes [I>=2σ (I)]	R_1 = 0.0548, wR_2 = 0.0958
Final R indexes [all data]	R_1 = 0.1196, wR_2 = 0.1147
Largest diff. peak/hole / e Å^{-3}	0.40/-0.55

Table S9 Bond Lengths for 2c.

Atom	Atom	Length/Å	Atom	Atom	Length/Å
Zn1	N1[1]	2.105(5)	N10	C3	1.336(7)
Zn1	N2	2.364(5)	N11	C12	1.341(6)
Zn1	N10	2.114(4)	N11	C16	1.330(7)
Zn1	N11	2.144(5)	N21	C22	1.334(6)
Zn1	N21	2.174(4)	N21	C26	1.344(6)
Zn1	N31	2.217(5)	N31	C32	1.345(6)
N1	C1	1.154(6)	N31	C36	1.332(7)
N2	C1	1.306(7)	C12	C13	1.376(7)
N2	C2	1.359(6)	C13	C14	1.375(7)
N3	N4	1.370(6)	C14	C15	1.375(7)
N3	C2	1.322(7)	C15	C16	1.361(8)
N4	N5	1.300(7)	C22	C23	1.376(7)
N5	N6	1.370(6)	C23	C24	1.375(7)
N6	C2	1.359(7)	C24	C25	1.391(7)
N6	C3	1.393(7)	C25	C26	1.360(7)
N7	N8	1.363(7)	C32	C33	1.378(7)
N7	C3	1.314(7)	C33	C34	1.363(8)
N8	N9	1.286(6)	C34	C35	1.376(7)
N9	N10	1.356(6)	C35	C36	1.379(7)

[1]1-X,1-Y,2-Z

Table S10 Bond Angles for 2c.

Atom	Atom	Atom	Angle/°	Atom	Atom	Atom	Angle/°
N1[1]	Zn1	N2	88.09(17)	C12	N11	Zn1	120.7(4)
N1[1]	Zn1	N10	167.71(18)	C16	N11	Zn1	121.8(4)
N1[1]	Zn1	N11	93.77(17)	C16	N11	C12	117.5(5)
N1[1]	Zn1	N21	88.81(16)	C22	N21	Zn1	120.3(3)
N1[1]	Zn1	N31	88.77(16)	C22	N21	C26	116.7(4)
N10	Zn1	N2	79.81(17)	C26	N21	Zn1	122.7(3)
N10	Zn1	N11	98.37(17)	C32	N31	Zn1	122.5(4)
N10	Zn1	N21	92.17(16)	C36	N31	Zn1	120.7(4)
N10	Zn1	N31	88.04(16)	C36	N31	C32	116.8(5)
N11	Zn1	N2	177.81(16)	N1	C1	N2	172.6(6)
N11	Zn1	N21	94.32(16)	N2	C2	N6	119.3(5)
N11	Zn1	N31	96.09(16)	N3	C2	N2	131.4(5)
N21	Zn1	N2	84.56(15)	N3	C2	N6	109.3(5)
N21	Zn1	N31	169.44(17)	N7	C3	N6	124.6(5)
N31	Zn1	N2	85.09(16)	N7	C3	N10	113.2(6)
C1	N1	Zn1[1]	152.4(4)	N10	C3	N6	122.2(5)
C1	N2	Zn1	116.6(3)	N11	C12	C13	122.2(5)
C1	N2	C2	117.5(5)	C14	C13	C12	119.2(5)
C2	N2	Zn1	115.5(3)	C13	C14	C15	118.8(5)
C2	N3	N4	105.1(5)	C16	C15	C14	118.5(5)
N5	N4	N3	112.1(5)	N11	C16	C15	123.8(5)
N4	N5	N6	105.7(4)	N21	C22	C23	124.2(5)
N5	N6	C3	123.1(4)	C24	C23	C22	118.2(5)
C2	N6	N5	107.7(5)	C23	C24	C25	118.4(5)
C2	N6	C3	129.1(5)	C26	C25	C24	119.4(5)
C3	N7	N8	103.3(5)	N21	C26	C25	123.0(5)
N9	N8	N7	110.4(5)	N31	C32	C33	123.7(6)
N8	N9	N10	109.4(5)	C34	C33	C32	118.3(5)
N9	N10	Zn1	128.1(3)	C33	C34	C35	119.3(5)
C3	N10	Zn1	127.8(4)	C34	C35	C36	118.9(5)
C3	N10	N9	103.6(4)	N31	C36	C35	123.0(5)

[1]1-X,1-Y,2-Z

Crystal structure determination of [2c]

Crystal Data for $C_{36}H_{30}N_{26}Zn_2$ (M =957.60 g/mol): monoclinic, space group $P2_1/n$ (no. 14), a = 9.580(5) Å, b = 13.076(8) Å, c = 15.904(9) Å, β = 97.636(13)°, V = 1974.5(19) Å^3, Z = 2, T = 100.03 K, μ(MoKα) = 1.283 mm^{-1}, $Dcalc$ = 1.611 g/cm^3, 8344 reflections measured (4.048° ≤ 2Θ ≤ 49.414°), 3376 unique (R_{int} = 0.0873, R_{sigma} = 0.1280) which were used in all calculations. The final R_1 was 0.0548 (I > 2σ(I)) and wR_2 was 0.1147 (all data).

Refinement model description

Number of restraints - 0

Details: No disorder was observed. Hydrogen atoms refined as riding models.
1. Fixed Uiso
At 1.2 times of:
All C(H) groups
2.a Aromatic/amide H refined with riding coordinates:
C12(H12), C13(H13), C14(H14), C15(H15), C16(H16), C22(H22), C23(H23), C24(H24), C25(H25), C26(H26), C32(H32), C33(H33), C34(H34), C35(H35), C36(H36)

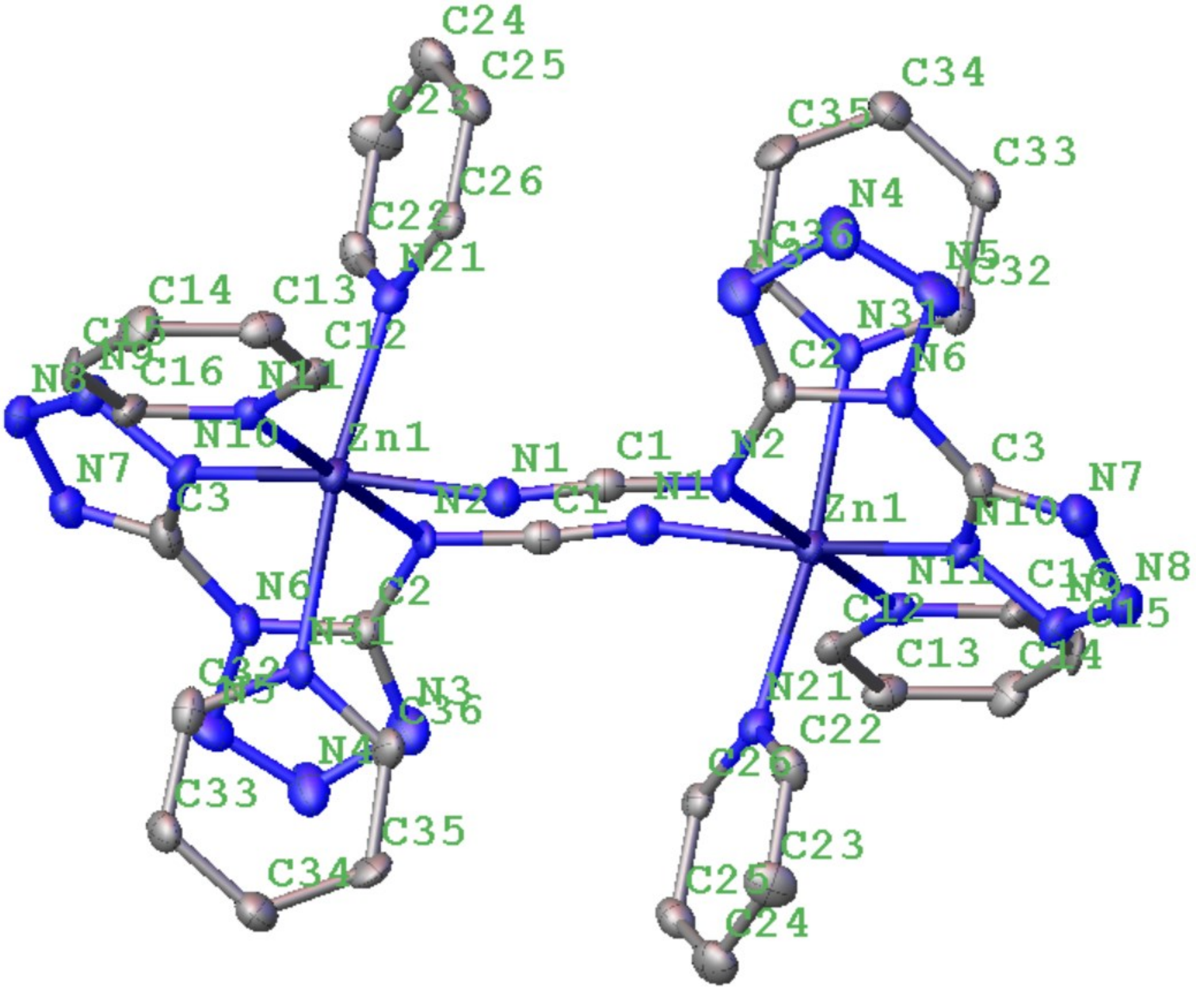

Figure S13. Thermal Ellipsoid plot of **2c**. Ellipsoids set at 50% probability. Hydrogen atoms omitted for clarity.

2d

Table S11 Crystal data and structure refinement for 2d.

Identification code	2d
Empirical formula	$C_{36}H_{30}N_{26}Cu_2$
Formula weight	953.94
Temperature/K	99.95
Crystal system	monoclinic
Space group	$P2_1/n$
a/Å	9.332(3)
b/Å	13.337(5)
c/Å	15.845(6)
α/°	90
β/°	97.844(7)
γ/°	90
Volume/Å^3	1953.8(12)
Z	2
ρ_{calc}g/cm^3	1.622
μ/mm^{-1}	1.157
F(000)	972.0
Crystal size/mm^3	0.81 × 0.42 × 0.01
Radiation	MoKα (λ = 0.71073)
2Θ range for data collection/°	4.008 to 49.636
Index ranges	$-10 \leq h \leq 10$, $-10 \leq k \leq 15$, $-18 \leq l \leq 18$
Reflections collected	9009
Independent reflections	3358 [R_{int} = 0.0723, R_{sigma} = 0.0900]
Data/restraints/parameters	3358/15/289
Goodness-of-fit on F^2	1.012
Final R indexes [I>=2σ (I)]	R_1 = 0.0471, wR_2 = 0.0804
Final R indexes [all data]	R_1 = 0.0915, wR_2 = 0.0923
Largest diff. peak/hole / e Å^{-3}	0.42/-0.72

Table S12 Bond Lengths for 2d.

Atom	Atom	Length/Å	Atom	Atom	Length/Å
Cu1	N1[1]	1.971(4)	N10	C3	1.344(5)
Cu1	N2	2.746(4)	N11	C12	1.353(5)
Cu1	N10	1.996(3)	N11	C16	1.339(5)
Cu1	N11	2.278(3)	N21	C22	1.337(5)
Cu1	N21	2.064(3)	N21	C26	1.350(5)
Cu1	N31	2.070(3)	N31	C32	1.358(5)
N1	C1	1.148(5)	N31	C36	1.332(5)
N2	C1	1.299(5)	C12	C13	1.376(6)
N2	C2	1.364(5)	C13	C14	1.380(6)
N3	N4	1.373(5)	C14	C15	1.381(6)
N3	C2	1.315(5)	C15	C16	1.376(6)
N4	N5	1.290(5)	C22	C23	1.387(5)
N5	N6	1.382(5)	C23	C24	1.378(6)
N6	C2	1.352(5)	C24	C25	1.381(6)
N6	C3	1.397(5)	C25	C26	1.375(6)
N7	N8	1.364(5)	C32	C33	1.375(6)
N7	C3	1.314(5)	C33	C34	1.374(6)
N8	N9	1.314(5)	C34	C35	1.384(6)
N9	N10	1.364(5)	C35	C36	1.380(6)

[1]1-X,1-Y,1-Z

Table S13 Bond Angles for 2d.

Atom	Atom	Atom	Angle/°	Atom	Atom	Atom	Angle/°
N1[1]	Cu1	N2	91.85(12)	C12	N11	Cu1	120.2(3)
N1[1]	Cu1	N10	167.55(14)	C16	N11	Cu1	123.7(3)
N1[1]	Cu1	N11	93.57(13)	C16	N11	C12	116.1(4)
N1[1]	Cu1	N21	89.36(13)	C22	N21	Cu1	122.1(3)
N1[1]	Cu1	N31	89.44(13)	C22	N21	C26	117.5(3)
N10	Cu1	N2	75.87(12)	C26	N21	Cu1	120.4(3)
N10	Cu1	N11	98.84(13)	C32	N31	Cu1	121.2(3)
N10	Cu1	N21	90.81(13)	C36	N31	Cu1	121.9(3)
N10	Cu1	N31	88.03(13)	C36	N31	C32	116.9(4)
N11	Cu1	N2	173.25(11)	N1	C1	N2	172.5(4)
N21	Cu1	N2	81.71(12)	N3	C2	N2	131.5(4)
N21	Cu1	N11	94.32(12)	N3	C2	N6	109.4(4)
N21	Cu1	N31	169.07(14)	N6	C2	N2	119.1(4)
N31	Cu1	N2	87.47(12)	N7	C3	N6	124.3(4)
N31	Cu1	N11	96.60(12)	N7	C3	N10	113.8(4)
C1	N1	Cu1[1]	158.5(3)	N10	C3	N6	121.8(4)
C1	N2	Cu1	110.0(3)	N11	C12	C13	123.7(4)
C1	N2	C2	119.8(4)	C12	C13	C14	118.7(4)
C2	N2	Cu1	104.3(2)	C13	C14	C15	118.7(4)
C2	N3	N4	105.0(3)	C16	C15	C14	118.8(4)
N5	N4	N3	112.6(3)	N11	C16	C15	123.9(4)
N4	N5	N6	105.0(3)	N21	C22	C23	122.8(4)
N5	N6	C3	122.3(3)	C24	C23	C22	118.9(4)
C2	N6	N5	107.9(3)	C23	C24	C25	118.8(4)
C2	N6	C3	129.7(3)	C26	C25	C24	119.0(4)
C3	N7	N8	103.1(3)	N21	C26	C25	122.9(4)
N9	N8	N7	111.0(3)	N31	C32	C33	123.2(4)
N8	N9	N10	108.2(3)	C34	C33	C32	118.8(4)
N9	N10	Cu1	124.6(3)	C33	C34	C35	118.9(4)
C3	N10	Cu1	131.4(3)	C36	C35	C34	118.8(4)
C3	N10	N9	103.9(3)	N31	C36	C35	123.3(4)

[1]1-X,1-Y,1-Z

Crystal structure determination of [2d]

Crystal Data for $C_{36}H_{30}N_{26}Cu_2$ (M =953.94 g/mol): monoclinic, space group $P2_1/n$ (no. 14), a = 9.332(3) Å, b = 13.337(5) Å, c = 15.845(6) Å, β = 97.844(7)°, V = 1953.8(12) Å^3, Z = 2, T = 99.95 K, μ(MoKα) = 1.157 mm^{-1}, $Dcalc$ = 1.622 g/cm^3, 9009 reflections measured (4.008° ≤ 2Θ ≤ 49.636°), 3358 unique (R_{int} = 0.0723, R_{sigma} = 0.0900) which were used in all calculations. The final R_1 was 0.0471 (I > 2σ(I)) and wR_2 was 0.0923 (all data).

Refinement model description

Number of restraints - 15

Details: There was no disorder, but the carbon atom of the carbodiimide possessed collapsing thermal parameters. We attribute this to charge buildup on the carbodiimide group due to this group being dissociated from the copper atom, leading to a larger electron density than the neutral carbon atom used for the crystallographic model. These thermal parameters were gently restrained.

1. Fixed Uiso
 At 1.2 times of:
  All C(H) groups
2. Rigid bond restraints
 N1, N2, C1
 with sigma for 1-2 distances of 0.005 and sigma for 1-3 distances of 0.005
3. Uiso/Uaniso restraints and constraints
N1 ≈ N2 ≈ C1: within 2A with sigma of 0.005 and sigma for terminal atoms of 0.01
4.a Aromatic/amide H refined with riding coordinates:
 C12(H12), C13(H13), C14(H14), C15(H15), C16(H16), C22(H22), C23(H23), C24(H24), C25(H25), C26(H26), C32(H32), C33(H33), C34(H34), C35(H35), C36(H36)

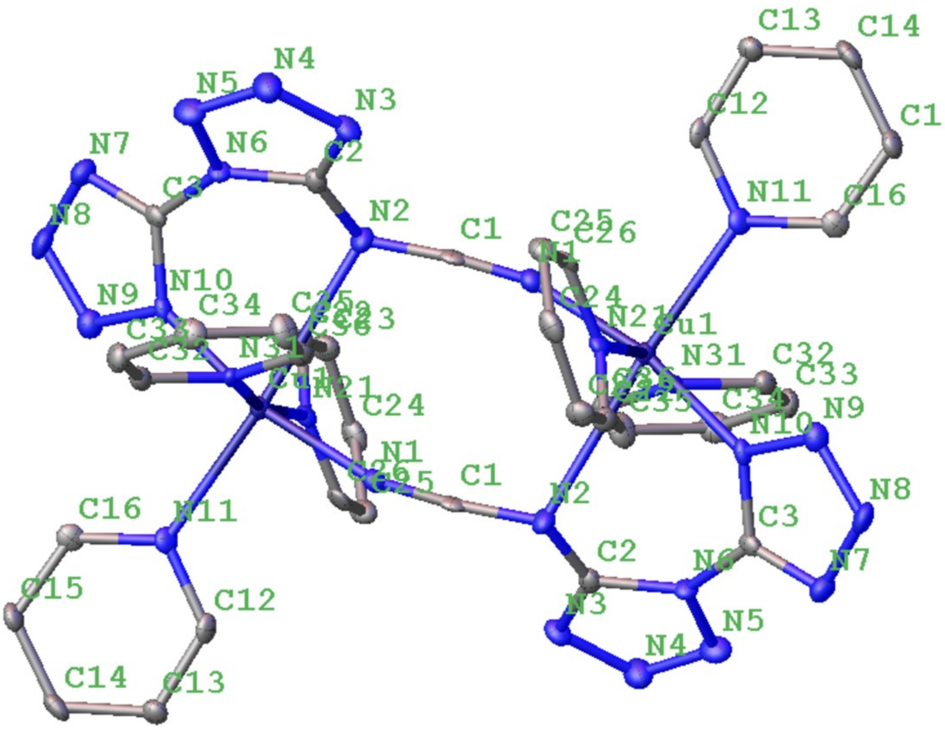

Figure S14. Thermal Ellipsoid plot of **2d**. Ellipsoids set at 50% probability. Hydrogen atoms omitted for clarity.

2e

Table S14 Crystal data and structure refinement for 2e

Identification code	2e
Empirical formula	$C_{44}H_{46}N_{26}O_2Ni_2$
Formula weight	1088.49
Temperature/K	100.09
Crystal system	monoclinic
Space group	$P2_1/c$
a/Å	11.927(2)
b/Å	20.144(4)
c/Å	10.560(2)
α/°	90
β/°	98.269(4)
γ/°	90
Volume/Å^3	2510.6(8)
Z	2
ρ_{calc}g/cm^3	1.440
μ/mm^{-1}	0.816
F(000)	1128.0
Crystal size/mm^3	0.17 × 0.075 × 0.02
Radiation	MoKα (λ = 0.71073)
2Θ range for data collection/°	3.45 to 56.046
Index ranges	$-15 \le h \le 15$, $-26 \le k \le 26$, $-12 \le l \le 13$
Reflections collected	22596
Independent reflections	6037 [R_{int} = 0.0459, R_{sigma} = 0.0489]
Data/restraints/parameters	6037/624/481
Goodness-of-fit on F^2	1.030
Final R indexes [I>=2σ (I)]	R_1 = 0.0461, wR_2 = 0.0932
Final R indexes [all data]	R_1 = 0.0809, wR_2 = 0.1055
Largest diff. peak/hole / e Å^{-3}	0.48/-0.40

Table S15 Bond Lengths for 2e.

Atom	Atom	Length/Å	Atom	Atom	Length/Å
Ni1	N1[1]	2.087(2)	C12	C13	1.377(8)
Ni1	N2	2.148(2)	C12A	C13A	1.379(7)
Ni1	N10	2.079(2)	C13	C14	1.379(8)
Ni1	N11	2.085(2)	C13A	C14A	1.379(6)
Ni1	N21	2.118(6)	C14	C15	1.375(8)
Ni1	N21A	2.123(8)	C14A	C15A	1.376(7)
Ni1	N31	2.097(2)	C15	C16	1.381(8)
N1	C1	1.159(3)	C15A	C16A	1.380(7)
N2	C1	1.309(3)	C22	C23	1.371(7)
N2	C2	1.352(3)	C22A	C23A	1.379(9)
N3	N4	1.365(3)	C23	C24	1.378(7)
N3	C2	1.324(3)	C23A	C24A	1.380(8)
N4	N5	1.297(3)	C24	C25	1.379(6)
N5	N6	1.366(3)	C24A	C25A	1.380(8)
N6	C2	1.351(3)	C25	C26	1.379(7)
N6	C3	1.403(3)	C25A	C26A	1.380(9)
N7	N8	1.353(3)	C32	C33	1.372(4)
N7	C3	1.315(3)	C33	C34	1.369(5)
N8	N9	1.309(3)	C34	C35	1.386(5)
N9	N10	1.359(3)	C35	C36	1.370(4)
N10	C3	1.331(3)	O1S	C1S	1.392(8)
N11	C12	1.339(8)	O1S	C4S	1.530(9)
N11	C12A	1.343(6)	C1S	C2S	1.495(7)
N11	C16	1.353(8)	C2S	C3S	1.564(8)
N11	C16A	1.349(6)	C3S	C4S	1.531(9)
N21	C22	1.345(7)	O1T	C1T	1.479(9)
N21	C26	1.335(7)	O1T	C4T	1.465(9)
N21A	C22A	1.348(8)	C1T	C2T	1.564(10)
N21A	C26A	1.349(9)	C2T	C3T	1.542(9)
N31	C32	1.342(4)	C3T	C4T	1.534(9)
N31	C36	1.331(3)			

[1]1-X,1-Y,1-Z

Table S16 Bond Angles for 2e.

Atom	Atom	Atom	Angle/°	Atom	Atom	Atom	Angle/°
N1[1]	Ni1	N2	87.28(8)	C26A	N21A	Ni1	118.5(11)
N1[1]	Ni1	N21	88.2(6)	C32	N31	Ni1	120.2(2)
N1[1]	Ni1	N21A	91.6(8)	C36	N31	Ni1	122.25(19)
N1[1]	Ni1	N31	89.04(9)	C36	N31	C32	117.6(3)
N10	Ni1	N1[1]	170.32(7)	N1	C1	N2	174.1(3)
N10	Ni1	N2	83.29(8)	N3	C2	N2	130.4(2)
N10	Ni1	N11	98.55(8)	N3	C2	N6	108.6(2)

Atom	Atom	Atom	Angle/°	Atom	Atom	Atom	Angle/°
N10	Ni1	N21	89.4(7)	N6	C2	N2	121.0(2)
N10	Ni1	N21A	86.4(10)	N7	C3	N6	124.3(2)
N10	Ni1	N31	93.27(9)	N7	C3	N10	114.1(2)
N11	Ni1	N1[1]	90.83(8)	N10	C3	N6	121.6(2)
N11	Ni1	N2	177.81(8)	N11	C12	C13	122.8(9)
N11	Ni1	N21	89.9(8)	N11	C12A	C13A	123.0(7)
N11	Ni1	N21A	87.7(12)	C12	C13	C14	118.8(9)
N11	Ni1	N31	90.32(8)	C14A	C13A	C12A	118.7(7)
N21	Ni1	N2	88.9(8)	C15	C14	C13	119.3(8)
N21A	Ni1	N2	91.2(12)	C15A	C14A	C13A	119.3(6)
N31	Ni1	N2	90.76(8)	C14	C15	C16	119.0(9)
N31	Ni1	N21	177.2(5)	C14A	C15A	C16A	118.8(6)
N31	Ni1	N21A	178.0(10)	N11	C16	C15	122.1(9)
C1	N1	Ni1[1]	143.5(2)	N11	C16A	C15A	122.8(7)
C1	N2	Ni1	120.27(16)	N21	C22	C23	121.2(6)
C1	N2	C2	117.2(2)	N21A	C22A	C23A	125.5(9)
C2	N2	Ni1	118.85(16)	C22	C23	C24	119.9(6)
C2	N3	N4	105.3(2)	C22A	C23A	C24A	117.9(9)
N5	N4	N3	112.3(2)	C23	C24	C25	119.6(6)
N4	N5	N6	105.1(2)	C23A	C24A	C25A	118.3(10)
N5	N6	C3	123.0(2)	C26	C25	C24	117.1(6)
C2	N6	N5	108.7(2)	C24A	C25A	C26A	120.1(10)
C2	N6	C3	128.2(2)	N21	C26	C25	124.0(7)
C3	N7	N8	103.2(2)	N21A	C26A	C25A	123.1(10)
N9	N8	N7	110.41(19)	N31	C32	C33	123.0(3)
N8	N9	N10	108.9(2)	C34	C33	C32	118.8(3)
N9	N10	Ni1	130.88(16)	C33	C34	C35	118.8(3)
C3	N10	Ni1	125.64(16)	C36	C35	C34	118.7(3)
C3	N10	N9	103.4(2)	N31	C36	C35	123.0(3)
C12	N11	Ni1	125.3(6)	C1S	O1S	C4S	98.0(7)
C12	N11	C16	117.9(8)	O1S	C1S	C2S	111.2(6)
C12A	N11	Ni1	117.0(5)	C1S	C2S	C3S	104.6(5)
C12A	N11	C16A	117.4(6)	C4S	C3S	C2S	99.3(6)
C16	N11	Ni1	116.2(6)	O1S	C4S	C3S	105.9(7)
C16A	N11	Ni1	125.5(5)	C4T	O1T	C1T	102.5(9)
C22	N21	Ni1	119.5(7)	O1T	C1T	C2T	101.8(9)
C26	N21	Ni1	122.2(7)	C3T	C2T	C1T	97.8(9)
C26	N21	C22	118.3(6)	C4T	C3T	C2T	105.6(7)
C22A	N21A	Ni1	126.2(11)	O1T	C4T	C3T	106.4(7)
C22A	N21A	C26A	115.2(9)				

[1]1-X,1-Y,1-Z

Crystal structure determination of [2e]

Crystal Data for $C_{44}H_{46}N_{26}O_2Ni_2$ (M =1088.49 g/mol): monoclinic, space group $P2_1/c$ (no. 14), a = 11.927(2) Å, b = 20.144(4) Å, c = 10.560(2) Å, β = 98.269(4)°, V = 2510.6(8) Å^3, Z = 2, T = 100.09 K, μ(MoKα) = 0.816 mm^{-1}, $Dcalc$ = 1.440 g/cm^3, 22596 reflections measured (3.45° ≤ 2Θ ≤ 56.046°), 6037 unique (R_{int} = 0.0459, R_{sigma} = 0.0489) which were used in all calculations. The final R_1 was 0.0461 (I > 2σ(I)) and wR_2 was 0.1055 (all data).

Refinement model description

Number of restraints – 624.

Details: Disorder in both bound pyridines and solvate THF was modeled with split atom models and restraints on distance and thermal parameters. Hydrogens were refined using a riding model.

1. Fixed Uiso
At 1.2 times of:
All C(H) groups, All C(H,H) groups
2. Restrained distances
C4T-C3T = C3T-C2T = C2T-C1T = C2S-C1S = C3S-C2S = C4S-C3S
1.54 with sigma of 0.01
O1S-C1S = O1S-C4S = O1T-C1T = O1T-C4T
1.45 with sigma of 0.01
N21A-C26A = N21-C26 = N21A-C22A = N21-C22
1.34 with sigma of 0.01
C26A-C25A = C26-C25 = C25-C24 = C25A-C24A = C24A-C23A = C24-C23 = C23A-C22A =
C23-C22
1.38 with sigma of 0.01
Ni1-N21A = Ni1-N21
2.085 with sigma of 0.01
N11-C16 = N11-C16A = N11-C12A = N11-C12
1.34 with sigma of 0.01
C16-C15 = C16A-C15A = C15-C14 = C15A-C14A = C14-C13 = C14A-C13A = C13-C12 = C13A-C12A
1.38 with sigma of 0.01
C4T-C1T
2.29 with sigma of 0.02
C4S-C1S
2.29 with sigma of 0.02
O1S-C3S
2.44 with sigma of 0.02
O1S-C2S
2.44 with sigma of 0.02
O1T-C2T
2.44 with sigma of 0.02
O1T-C3T
2.44 with sigma of 0.02
C1T-C3T
2.52 with sigma of 0.02
C2T-C4T
2.52 with sigma of 0.02
C1S-C3S

2.52 with sigma of 0.02
C2S-C4S
2.52 with sigma of 0.02
C22-C26
2.32 with sigma of 0.04
C22A-C26A
2.32 with sigma of 0.04
N21A-C23A
2.36 with sigma of 0.04
N21A-C25A
2.36 with sigma of 0.04
N21-C23
2.36 with sigma of 0.04
N21-C25
2.36 with sigma of 0.04
C22A-C24A
2.39 with sigma of 0.04
C23A-C25A
2.39 with sigma of 0.04
C24A-C26A
2.39 with sigma of 0.04
C22-C24
2.39 with sigma of 0.04
C23-C25
2.39 with sigma of 0.04
C24-C26
2.39 with sigma of 0.04
C16-C14
2.39 with sigma of 0.02
C15-C13
2.39 with sigma of 0.02
C14-C12
2.39 with sigma of 0.02
C16A-C14A
2.39 with sigma of 0.02
C15A-C13A
2.39 with sigma of 0.02
C14A-C12A
2.39 with sigma of 0.02
N11-C15
2.36 with sigma of 0.02
N11-C13
2.36 with sigma of 0.02
N11-C15A
2.36 with sigma of 0.02
N11-C13A
2.36 with sigma of 0.02
C16-C12
2.32 with sigma of 0.02
C16A-C12A

2.32 with sigma of 0.02
3. Rigid bond restraints
O1S, C1S, C2S, C3S, C4S, O1T, C1T, C2T, C3T, C4T
with sigma for 1-2 distances of 0.01 and sigma for 1-3 distances of 0.01
N21, N21A, C22, C22A, C23, C23A, C24, C24A, C25, C25A, C26, C26A
with sigma for 1-2 distances of 0.01 and sigma for 1-3 distances of 0.01
N11, C12, C12A, C13, C13A, C14, C14A, C15, C15A, C16, C16A
with sigma for 1-2 distances of 0.01 and sigma for 1-3 distances of 0.01
4. Uiso/Uaniso restraints and constraints
O1S ≈ C1S ≈ C2S ≈ C3S ≈ C4S ≈ O1T ≈ C1T ≈ C2T ≈
C3T ≈ C4T: within 2A with sigma of 0.04 and sigma for terminal atoms of 0.08
N21 ≈ N21A ≈ C22 ≈ C22A ≈ C23 ≈ C23A ≈ C24 ≈ C24A
≈ C25 ≈ C25A ≈ C26 ≈ C26A: within 2A with sigma of 0.01 and
sigma for terminal atoms of 0.02
N11 ≈ C12 ≈ C12A ≈ C13 ≈ C13A ≈ C14 ≈ C14A ≈ C15
≈ C15A ≈ C16 ≈ C16A: within 2A with sigma of 0.01 and sigma for
terminal atoms of 0.02
5. Others

Sof(O1T)=Sof(C1T)=Sof(H1TA)=Sof(H1TB)=Sof(C2T)=Sof(H2TA)=Sof(H2TB)=Sof(C3T
)=
Sof(H3TA)=Sof(H3TB)=Sof(C4T)=Sof(H4TA)=Sof(H4TB)=1-FVAR(1)

Sof(O1S)=Sof(C1S)=Sof(H1SA)=Sof(H1SB)=Sof(C2S)=Sof(H2SA)=Sof(H2SB)=Sof(C3S)
=
Sof(H3SA)=Sof(H3SB)=Sof(C4S)=Sof(H4SA)=Sof(H4SB)=FVAR(1)
Sof(N21A)=Sof(C22A)=Sof(H22A)=Sof(C23A)=Sof(H23A)=Sof(C24A)=Sof(H24A)=
Sof(C25A)=Sof(H25A)=Sof(C26A)=Sof(H26A)=1-FVAR(2)
Sof(N21)=Sof(C22)=Sof(H22)=Sof(C23)=Sof(H23)=Sof(C24)=Sof(H24)=Sof(C25)=
Sof(H25)=Sof(C26)=Sof(H26)=FVAR(2)
Sof(C12A)=Sof(H12A)=Sof(C13A)=Sof(H13A)=Sof(C14A)=Sof(H14A)=Sof(C15A)=
Sof(H15A)=Sof(C16A)=Sof(H16A)=1-FVAR(3)
Sof(C12)=Sof(H12)=Sof(C13)=Sof(H13)=Sof(C14)=Sof(H14)=Sof(C15)=Sof(H15)=
Sof(C16)=Sof(H16)=FVAR(3)
6.a Secondary CH2 refined with riding coordinates:
C1S(H1SA,H1SB), C2S(H2SA,H2SB), C3S(H3SA,H3SB), C4S(H4SA,H4SB), C1T(H1TA,
H1TB), C2T(H2TA,H2TB), C3T(H3TA,H3TB), C4T(H4TA,H4TB)
6.b Aromatic/amide H refined with riding coordinates:
C12(H12), C12A(H12A), C13(H13), C13A(H13A), C14(H14), C14A(H14A), C15(H15),
C15A(H15A), C16(H16), C16A(H16A), C22(H22), C22A(H22A), C23(H23),
C23A(H23A),
C24(H24), C24A(H24A), C25(H25), C25A(H25A), C26(H26), C26A(H26A), C32(H32),
C33(H33), C34(H34), C35(H35), C36(H36)

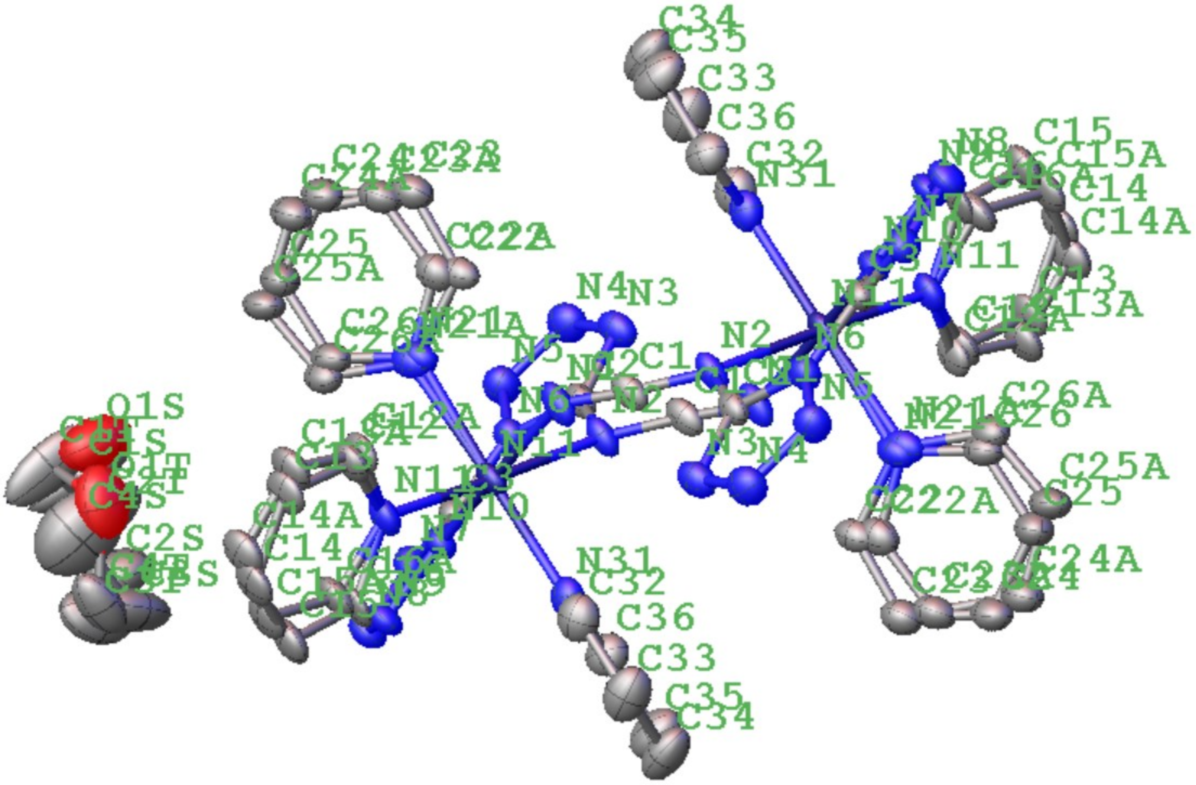

Figure S15. Thermal Ellipsoid plot of **2e**·THF. Ellipsoids set at 50% probability. Hydrogen atoms omitted for clarity.

3

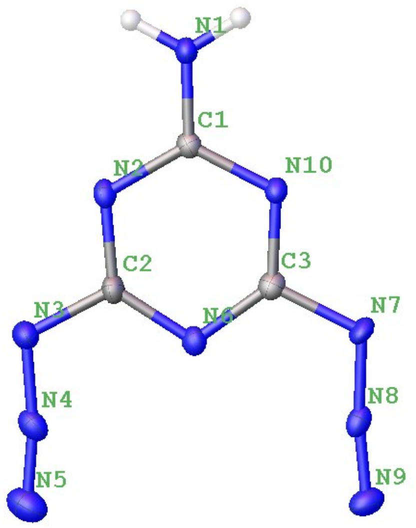

Figure S16. Thermal ellipsoid plot of **3**. Ellipsoids at 50% probability level.

Table S13 Crystal data and structure refinement for 3.

Identification code	3
Empirical formula	$C_3H_2N_{10}$
Formula weight	178.15
Temperature/K	99.99
Crystal system	monoclinic
Space group	$P2_1/n$
a/Å	8.018(4)
b/Å	4.669(3)
c/Å	18.877(10)
α/°	90
β/°	101.238(10)
γ/°	90
Volume/Å^3	693.1(6)
Z	4
ρ_{calc}g/cm^3	1.707
μ/mm^{-1}	0.134
F(000)	360.0
Crystal size/mm^3	0.3 × 0.07 × 0.042
Radiation	MoKα (λ = 0.71073)
2Θ range for data collection/°	4.4 to 50.498
Index ranges	$-9 \le h \le 9$, $-5 \le k \le 5$, $0 \le l \le 22$
Reflections collected	1241
Independent reflections	1241 [R_{int} = ?, R_{sigma} = 0.0378]
Data/restraints/parameters	1241/0/125
Goodness-of-fit on F^2	1.098
Final R indexes [I>=2σ (I)]	R_1 = 0.0617, wR_2 = 0.1711
Final R indexes [all data]	R_1 = 0.0772, wR_2 = 0.1795
Largest diff. peak/hole / e Å^{-3}	0.43/-0.35

Table S14 Bond Lengths for 3.

Atom	Atom	Length/Å	Atom	Atom	Length/Å
N7	N8	1.259(4)	N2	C1	1.354(4)
N7	C3	1.395(4)	N10	C3	1.320(4)
N6	C3	1.331(5)	N10	C1	1.367(4)
N6	C2	1.333(5)	N1	C1	1.302(4)
N3	C2	1.381(5)	N9	N8	1.117(4)
N3	N4	1.254(4)	N4	N5	1.118(5)
N2	C2	1.322(5)			

Table S15 Bond Angles for 3.

Atom	Atom	Atom	Angle/°	Atom	Atom	Atom	Angle/°
N8	N7	C3	114.1(3)	N10	C3	N6	128.3(3)
C3	N6	C2	112.2(3)	N6	C2	N3	119.8(3)
N4	N3	C2	113.8(3)	N2	C2	N6	127.2(3)
C2	N2	C1	115.3(3)	N2	C2	N3	113.1(3)
C3	N10	C1	114.1(3)	N5	N4	N3	171.5(4)
N9	N8	N7	172.5(3)	N2	C1	N10	122.9(3)
N6	C3	N7	118.4(3)	N1	C1	N2	118.8(3)
N10	C3	N7	113.3(3)	N1	C1	N10	118.4(3)

Crystal structure determination of 3

Crystal Data for $C_3H_2N_{10}$ (M =178.15 g/mol): monoclinic, space group P2$_1$/n (no. 14), a = 8.018(4) Å, b = 4.669(3) Å, c = 18.877(10) Å, β = 101.238(10)°, V = 693.1(6) Å^3, Z = 4, T = 99.99 K, μ(MoKα) = 0.134 mm^{-1}, $Dcalc$ = 1.707 g/cm^3, 1241 reflections measured (4.4° ≤ 2Θ ≤ 50.498°), 1241 unique (R_{int} = ?, R_{sigma} = 0.0378) which were used in all calculations. The final R_1 was 0.0617 (I > 2σ(I)) and wR_2 was 0.1795 (all data).

Refinement model description

Number of restraints - 0, number of constraints - 0.

Details: This crystal was diagnosed as a partial merohedral twin using TwinRotMat (PLATON), and was refined using an HKLF5 file. Amine hydrogen positions were refined. Thermal paramters were refined using a riding model. All other hydrogens were refined using a riding model.

1. Twinned data refinement
 Scales: 0.878(6)
 0.122(6)
2. Fixed Uiso
 At 1.2 times of:
  All N(H,H) groups

6. Raw quantum chemical data:

Optimized geometries, electronic energies, and thermal corrections to thermodynamic potentials of all compounds under study

M06-2X/6-311++G(2df,p) geometries, zero-point vibrational energies (unscaled), thermal corrections to thermodynamic potentials, free energies of solvation, and DLPNO-CCSD(T)/aug-cc-pVQZ electronic energies. The species are named in accordance with Figure 2 from the manuscript.

Reagent complex (nitrene+Zn)

Zero-point correction= 0.065630 (Hartree/Particle)
Thermal correction to Energy= 0.077092
Thermal correction to Enthalpy= 0.078036
Thermal correction to Gibbs Free Energy= 0.025998

Electronic energy:

M06-2X = -2440.9200448
DLPNO-CCSD(T)= -2439.591214

PCM energy (electronic energy+solvation):

M06-2X = -2440.9926961

Center Number	Atomic Number	Atomic Type	Coordinates (Angstroms) X	Y	Z
1	7	0	2.307562	0.218681	0.000000
2	7	0	0.000000	0.656150	0.000000
3	7	0	-2.320099	1.042765	0.000000
4	7	0	-1.558024	-1.180108	0.000000
5	7	0	0.818353	-1.538247	0.000000
6	7	0	2.583542	2.547572	0.000000
7	7	0	2.413429	1.445868	0.000000
8	6	0	0.968828	-0.238734	0.000000
9	6	0	-1.364834	0.175183	0.000000
10	6	0	-0.485403	-1.918716	0.000000
11	7	0	-0.729968	-3.296144	0.000000
12	7	0	0.295823	-3.999514	0.000000
13	7	0	1.129506	-4.731861	0.000000
14	30	0	-0.976414	2.457916	0.000000

TS1

Zero-point correction= 0.063343 (Hartree/Particle)
Thermal correction to Energy= 0.074948
Thermal correction to Enthalpy= 0.075892
Thermal correction to Gibbs Free Energy= 0.022979

Electronic energy:

M06-2X = -2440.9014888
DLPNO-CCSD(T)= -2439.570247

PCM energy (electronic energy+solvation):

M06-2X = -2440.9648338

Center Number	Atomic Number	Atomic Type	Coordinates (Angstroms) X	Y	Z
1	7	0	2.353387	0.425692	0.000000
2	7	0	0.000000	0.785555	0.000000
3	7	0	-2.606495	0.824108	0.000000
4	7	0	-1.529384	-1.343759	0.000000
5	7	0	0.894557	-1.352499	0.000000
6	7	0	2.632039	2.755882	0.000000
7	7	0	2.466004	1.654593	0.000000
8	6	0	0.996548	-0.012186	0.000000
9	6	0	-1.763350	-0.065619	0.000000
10	6	0	-0.319760	-1.884678	0.000000
11	7	0	-0.388749	-3.284233	0.000000
12	7	0	0.722958	-3.845391	0.000000
13	7	0	1.647667	-4.456967	0.000000
14	30	0	-1.227484	2.221135	0.000000

I1

Zero-point correction= 0.064393 (Hartree/Particle)
Thermal correction to Energy= 0.076577
Thermal correction to Enthalpy= 0.077521
Thermal correction to Gibbs Free Energy= 0.023584

Electronic energy:

M06-2X = -2440.9341841
DLPNO-CCSD(T)= -2439.604087

PCM energy (electronic energy+solvation):

M06-2X = -2440.9798518

Center Number	Atomic Number	Atomic Type	Coordinates (Angstroms) X	Y	Z
1	7	0	0.696588	2.390697	0.267940
2	7	0	1.555877	0.312790	-0.413662
3	7	0	0.259230	-2.939255	0.262899
4	7	0	-1.075914	-1.156387	1.272332
5	7	0	-0.831497	0.772430	-0.142475
6	7	0	2.920184	3.135626	0.383909
7	7	0	1.882592	2.744436	0.317751
8	6	0	0.548616	1.012682	-0.102638
9	6	0	-0.433633	-2.091960	0.766684
10	6	0	-1.514763	-0.122518	0.443367
11	7	0	-2.916043	-0.138379	0.329186
12	7	0	-3.361835	0.823134	-0.328142
13	7	0	-3.850946	1.638421	-0.896508
14	30	0	1.381701	-1.529127	-0.467236

TS2

Zero-point correction= 0.063482 (Hartree/Particle)
Thermal correction to Energy= 0.074888
Thermal correction to Enthalpy= 0.075832
Thermal correction to Gibbs Free Energy= 0.023738

Electronic energy:

M06-2X = -2440.9057018
DLPNO-CCSD(T)= -2439.580555

PCM energy (electronic energy+solvation):

M06-2X = -2440.9568923

Center Number	Atomic Number	Atomic Type	Coordinates (Angstroms) X	Y	Z
1	7	0	0.153174	2.549283	0.447378
2	7	0	1.361807	0.736611	-0.362739
3	7	0	0.908312	-2.704771	0.182900
4	7	0	-0.854165	-1.336973	1.181523
5	7	0	-1.060959	0.679393	-0.108889
6	7	0	2.389578	2.497882	0.136619
7	7	0	1.371976	2.936852	0.425207
8	6	0	0.225143	1.205350	-0.018048
9	6	0	0.012248	-2.061697	0.673107
10	6	0	-1.519215	-0.386441	0.406155
11	7	0	-2.875562	-0.719192	0.267946
12	7	0	-3.532324	0.152918	-0.336782
13	7	0	-4.199447	0.864736	-0.860793
14	30	0	1.735141	-1.071348	-0.439129

I2

Zero-point correction= 0.066390 (Hartree/Particle)
Thermal correction to Energy= 0.077638
Thermal correction to Enthalpy= 0.078582
Thermal correction to Gibbs Free Energy= 0.026727

Electronic energy:

M06-2X = -2440.9604981
DLPNO-CCSD(T)= -2439.631364

PCM energy (electronic energy+solvation):

M06-2X = -2441.0259897

Center Number	Atomic Number	Atomic Type	Coordinates (Angstroms) X	Y	Z
1	7	0	0.014563	2.616222	0.341199
2	7	0	1.237806	0.891117	-0.144299
3	7	0	1.173058	-2.559051	-0.011878
4	7	0	-0.798565	-1.494254	0.955619
5	7	0	-1.226197	0.654153	-0.043793
6	7	0	2.048833	1.962792	0.018461
7	7	0	1.310774	2.963441	0.310866
8	6	0	-0.032845	1.328508	0.075370
9	6	0	0.173513	-2.052610	0.445771
10	6	0	-1.562641	-0.512209	0.328858
11	7	0	-2.886847	-0.948566	0.192483
12	7	0	-3.641630	-0.072392	-0.280121
13	7	0	-4.387934	0.632194	-0.694255
14	30	0	1.954160	-0.836724	-0.320332

TS3
Zero-point correction= 0.065331 (Hartree/Particle)
Thermal correction to Energy= 0.075786
Thermal correction to Enthalpy= 0.076730
Thermal correction to Gibbs Free Energy= 0.026806
Electronic energy:
M06-2X = -2440.925072
DLPNO-CCSD(T)= -2439.601112
PCM energy (electronic energy+solvation):
M06-2X = -2440.9917174

Center Number	Atomic Number	Atomic Type	Coordinates (Angstroms) X	Y	Z
1	7	0	-0.367332	2.541644	0.344416
2	7	0	1.071115	0.972798	-0.094980
3	7	0	1.356213	-2.484481	-0.069794
4	7	0	-0.771637	-1.738868	0.865013
5	7	0	-1.324510	0.420720	-0.034779
6	7	0	1.740181	2.136552	0.076653
7	7	0	0.879117	3.043693	0.332370
8	6	0	-0.245408	1.258363	0.091387
9	6	0	0.286607	-2.131119	0.371038
10	6	0	-1.601294	-0.794017	0.288981
11	7	0	-2.901687	-1.232339	0.104667
12	7	0	-3.518072	-0.195364	-0.379117
13	7	0	-3.200956	0.873685	-0.641768
14	30	0	1.954118	-0.678855	-0.267574

P1
Zero-point correction= 0.068461 (Hartree/Particle)
Thermal correction to Energy= 0.078611
Thermal correction to Enthalpy= 0.079556
Thermal correction to Gibbs Free Energy= 0.030668
Electronic energy:
M06-2X = -2440.965787
DLPNO-CCSD(T)= -2439.639976
PCM energy (electronic energy+solvation):
M06-2X = -2441.040046

Center Number	Atomic Number	Atomic Type	Coordinates (Angstroms) X	Y	Z
1	7	0	-0.499793	2.497945	0.448520
2	7	0	0.951302	0.994491	-0.162587
3	7	0	1.397406	-2.437963	0.026401
4	7	0	-0.719894	-1.715395	1.005154
5	7	0	-1.409691	0.351095	-0.079969
6	7	0	1.605633	2.149662	0.092420
7	7	0	0.739858	3.023630	0.436444
8	6	0	-0.349491	1.253792	0.087666
9	6	0	0.327421	-2.108962	0.484695
10	6	0	-1.638865	-0.931962	0.342181
11	7	0	-2.862562	-1.249782	0.043109
12	7	0	-3.393212	-0.163474	-0.569817
13	7	0	-2.564714	0.789611	-0.649268
14	30	0	1.908509	-0.631865	-0.320670
